# Risk of new-onset and recurrent uveitis with different biologics for ankylosing spondylitis: a network meta-analysis

**DOI:** 10.3389/fimmu.2025.1556313

**Published:** 2025-06-20

**Authors:** Xu Zhao, Qingqing Xie, Xinyi He, Yiwei Lu, Menglan Li, Shiquan Shuai

**Affiliations:** ^1^ Department of Rheumatology and Immunology, Beijing Anzhen Nanchong Hospital of Capital Medical University, Nanchong Central Hospital, Sichuan, Nanchong, China; ^2^ The Second Clinical Medical College of North Sichuan Medical College, Sichuan, Nanchong, China; ^3^ Nanchong Key Laboratory of Inflammation and Immunization, Beijing Anzhen Nanchong Hospital of Capital Medical University (Nanchong Central Hospital), Sichuan, Nanchong, China

**Keywords:** TNF-α inhibitor, IL-17 inhibitor, JAK inhibitor, ankylosing spondylitis, uveitis

## Abstract

**Background:**

Uveitis is a common extra-articular manifestation of ankylosing spondylitis (AS), and a systematic analysis of the effects of biologics on new-onset and recurrent uveitis is clinically important.

**Methods:**

We conducted a network meta-analysis (NMA) to assess the impact of anti-TNF-α (adalimumab, etanercept, golimumab, and infliximab), IL-17 inhibitors (secukinumab, bimekizumab, and ixekizumab), and JAK inhibitors (tofacitinib and upadacitinib) on new-onset and recurrent uveitis. Phase II/III double-blind randomized controlled trials and cohort studies were included. The relative risk (RR) was estimated, and drug efficacy was ranked based on the surface under the cumulative ranking curve (SUCRA).

**Results:**

A total of 17 articles with 18 studies and 11,529 AS patients were included. For new-onset uveitis, adalimumab reduced the risk significantly compared to etanercept and golimumab (RR: 0.30, 0.61), while etanercept increased the risk compared to golimumab and infliximab (RR: 2.03, 2.47). The SUCRA demonstrated that upadacitinib (84.0%) exhibited better efficacy, while ixekizumab (8.7%) was less effective than placebo (29.9%). For recurrent uveitis, adalimumab significantly reduced the risk compared to etanercept (RR: 0.70), while etanercept increased the risk compared to golimumab and infliximab (RR: 1.37, 1.70). Bimekizumab 160 mg and 320 mg were the most efficacious (SUCRA: 83.9%, 83.5%). A comprehensive analysis revealed that bimekizumab 320 mg and 160 mg were the most effective in reducing the incidence of uveitis. Ixekizumab and secukinumab were less effective than placebo.

**Conclusion:**

JAK inhibitors were more effective for new-onset uveitis in AS patients. Inhibition of IL-17A (secukinumab and ixekizumab) alone might increase the risk of uveitis, while simultaneous inhibition of IL-17A and IL-17F (bimekizumab) significantly reduced the risk. Etanercept increased the risk of uveitis compared to other TNF-α inhibitors.

## 1 Introduction

Ankylosing spondylitis (AS) is a chronic inflammatory disease that mainly affects the axial skeleton. AS is classified as spinal arthropathy (1). Its primary clinical manifestations are chronic inflammatory lower back pain and stiffness. These symptoms are relieved after exercise but show no significant improvement after rest (2). Uveitis is one of the most common extra-articular complications of AS. It can be classified into the following categories based on the site of inflammation: anterior uveitis(AU), intermediate uveitis, posterior uveitis, and panuveitis. AU shares a significant genetic risk factor with AS—the HLA-B*27 gene, whose epitope likely plays a crucial role in the immunologically mediated pathogenesis of the disease (3–5). Epidemiological studies show that about 20% to 40% of AS patients experience uveitis at least once during the course of their disease, and the incidence increases with the progression of AS (6, 7). It is important to recognize that uveitis is not merely a local inflammatory manifestation; it also serves as a marker of systemic inflammation. Furthermore, it is significantly associated with an elevated risk of cardiovascular diseases, including stroke and major cardiovascular events, in patients with AS (8, 9). Clinically, approximately 30% of uveitis patients require biologics to control the disease. This can translate to increased treatment costs, and in some refractory cases, may lead to vision impairment due to the chronic nature of the disease (10, 11).

In recent years, biologics have been widely applied in treating AS. They have significantly improved the symptoms, vital signs, and quality of life in AS patients by targeting the inhibition of inflammatory pathways, including tumor necrosis factor-α (TNF-α), interleukin-17 (IL-17), and Janus kinase (JAK) (12). However, the results of existing studies are still inconclusive regarding the effect of biologics in reducing new-onset and recurrent uveitis in AS patients. A large observational study reported that the incidence of uveitis in patients receiving adalimumab, infliximab, and etanercept (per 100 patient-years) were 13.6, 27.5, and 60.3, respectively, while the pre-treatment incidence of uveitis was 36.8, 45.5, and 41.6, respectively. The results indicated that adalimumab and infliximab reduced the risk of AU, while etanercept increased the risk of AU (13). Furthermore, another study corroborated that etanercept markedly elevated the likelihood of new-onset and recurrent AU in comparison to anti-TNF-α monoclonal antibodies (mAbs) (14).

Given the wide variety of biologics, the differences in their mechanisms of action and effect profiles, and the fact that previous studies largely focused on a single drug or a particular class of drugs, it is imperative to comprehensively assess the overall effect of different biologics in the management of AS-related uveitis. Accordingly, the current study employed a systematic network meta-analysis (NMA) to evaluate and quantify the impact of various biologics on new-onset and recurrent uveitis in AS patients. The objective was to furnish clinicians with evidence-based guidance for the management of AS-related uveitis, promoting personalized and precise treatment.

## 2 Materials and methods

The current study was implemented in accordance with the PRISMA reporting guideline (15) and was registered in the international prospective register of systematic reviews, Prospero (registration number: CRD42024588996). The ethical approval was not required according to Health Research Agency (HRA) guidelines. Data were available upon reasonable request. The PRISMA list is provided in Attachment 1.

### 2.1 Retrieval strategy

Two independent researchers (Xu Zhao and Xinyi He) carried out a comprehensive search across PubMed, Embase, the Cochrane Library, and Web of Science with a period from the database establishment up to August 12, 2024. Search terms consisted of subject terms and free terms, such as spondylitis, ankylosing”, “etanercept”, “adalimumab”, “infliximab”, “bimekizumab”, “golimumab”, “secukinumab”, “upadacitinib”, “tofacitinib”, and “tumor necrosis factor inhibitors”. Details are presented in Attachment 2.

### 2.2 Eligibility criteria

Inclusion criteria were as follows: Randomized controlled trials (RCTs) and cohort studies (Cohort) that complied with PICOS were included. The study subjects were AS patients who met the revised New York criteria (16); interventions included etanercept, adalimumab, infliximab, or other biologics; the control group received a placebo or other biologics. Open-label controlled studies were only included when there was an initial double-blind period and detailed safety analysis, and there was no limit on the length of follow-up; outcome indicators were new-onset and recurrent uveitis.

Exclusion criteria were as follows: Reviews, meta-analyses (MAs), case reports, animal experiments, conference abstracts, conference papers, guidelines, letters, non-English literature, studies that did not report outcome indicators of interesting, and literature that did not meet other intervention types.

### 2.3 Literature screening

Two researchers (Xu Zhao and Xinyi He) independently screened the literature based on the titles and abstracts in Endnote, and then assessed the full text of the remaining studies to confirm whether they met the inclusion criteria. In case of any disagreement, a third researcher, Qingqing Xie, made the final decision.

### 2.4 Data extraction

Two researchers (Xu Zhao and Xinyi He) independently extracted relevant data. In case of any disagreement, a third researcher, Qingqing Xie, made the final decision. The extracted contents included author, publication year, study country or region, study design, type of biologics, total sample size included, age, gender ratio, dose, double-blind period and open-label period of RCT, history of uveitis, types of uveitis, and the number of new-onset and recurrent uveitis.

### 2.5 Quality assessment

Study quality and risk of bias (ROB) were evaluated independently by two researchers (Xu Zhao and Xinyi He) via the RCT ROB assessment tool 2.0 (ROB2. 0) (17) and the Newcastle-Ottawa Scale (NOS) (18). A third researcher (Qingqing Xie) assisted in resolving discrepancies in the process. ROB2.0 assessment items included randomization bias, established intervention bias, missing outcome data bias, outcome measurement bias, and selective reporting bias. The studies were categorized as low risk, some concern, and high risk. The evaluation by NOS was based on 3 aspects: cohort selection, comparability, and outcome measurement. The highest score was 9 stars, and a study with a score of more than 6 stars was considered high-quality.

### 2.6 Statistical analysis

Statistical analysis was carried out via Stata 15 and R (version 4.2.2), and the Monte Carlo Markov chain (MCMC) was utilized in the Bayesian framework for the NMA. The model was run with four chains, including 20,000 annealing iterations, and completed after a total of 80,000 simulation iterations. The relative risk (RR) was applied as the effect size, and the combined effect size was calculated. Publication bias was assessed via a funnel plot. I^2^ was adopted to quantitatively analyze the heterogeneity between the results of each study, with a value distributed between 0% and 100%. 0% indicated no heterogeneity, and a higher I^2^ represented a greater heterogeneity. The consistency test was used to assess the agreement between direct and indirect evidence. Dots in the evidence network diagram represented various drugs, and lines between the dots represented a direct comparison between two drugs. If there were no lines between two drugs, it indicated an indirect comparison. The surface under the cumulative ranking curve (SUCRA) was employed to rank the efficacy of different treatments, and the best overall treatment was identified by SUCRA for new onsets and recurrences. The results of multiple comparisons were displayed directly via a league table to clarify the effect values of different interventions.

## 3 Results

### 3.1 Results of literature screening

**Figure 1:**
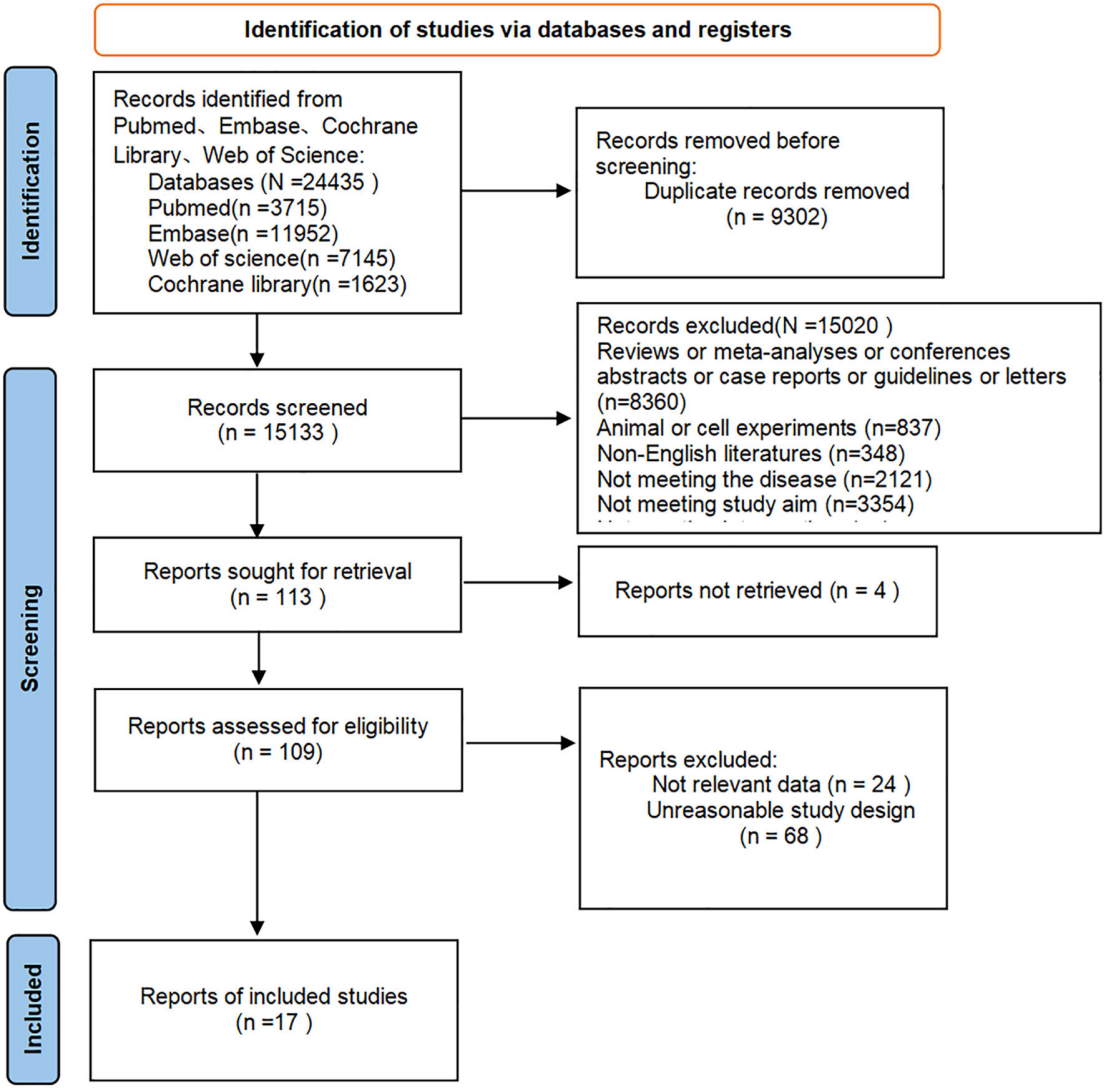
The literature screening process.

A total of 24,435 articles were retrieved, and after excluding 9,302 duplicates, 15,133 articles remained. Following preliminary screening by titles and abstracts, 113 articles were potentially eligible. After further screening by full texts, 17 articles (12 RCTs and 5 cohort studies) were finally included. The literature screening process is shown in **Figure 1**.

### 3.2 Baseline data

The 17 articles encompassed 18 independent studies, with 11,529 patients and a patient age ranging from 38.1 ± 16.1 to 46.1 ± 12.4 years. The proportion of male patients ranged from 67.8% to 84.5%. These studies covered more than 100 regions worldwide and the study drugs included adalimumab, bimekizumab, etanercept, golimumab, infliximab, secukinumab, tofacitinib, upadacitinib, and ixekizumab. Baseline characteristics are shown in **Table 1**.

**Table 1:** Baseline characteristics.

Baseline Data														
ID	Author	Year	Country	Design	Total	Age (Mean+SD)	Male (%)	Types of Uveitis	Drugs	Patients	History of uveitis	New-onset uveitis	Recurrent uveitis	Follow-up time
1	Heijde (40)	2020	74 sites across 10 countries	RCT	303	42.2 ± 11.8	256 (84.5)	AU	Bimekizumab 16 mg	61	46	0	0	12 weeks
									Bimekizumab 64 mg	61		0	0	
									Bimekizumab 160 mg	60		0	0	
									Bimekizumab 320 mg	61		0	0	
									Placebo	60		0	0	
2	Baraliakos (41)	2022	74 sites across 10 countries	RCT	299	42.2 ± 11.8	256 (84.5)	AU	Bimekizumab 160 mg	149	46	0	1	36 weeks
									Bimekizumab 320 mg	150		0	1	
3	Deodhar (42)	2021	75 centers in 14 countries	RCT	269	41.1 ± 11.5	224 (83.3)	uveitis	Tofacitinib	133	22	0	1	16 weeks
									Placebo	136	20	0	3	
4	Heijde (43)	2022	119 sites in 22 countries	RCT	420	42.4 ± 12.1	311 (74.0)	uveitis	Upadacitinib	211	21	0	1	14 weeks
									Placebo	209	15	1	2	
5	Heijde (44)	2019	62 centers in 20 countries	RCT	187	45.3 ± 12.5	132 (70.6)	uveitis	Upadacitinib	93	NR	0	0	14 weeks
									Placebo	94		0	0	
6A	Baeten (29)	2015	106 centers across Eurasia-America	RCT	371	41.8 ± 12.4	257 (69.3)	uveitis	Secukinumab	249	40	1	5	52 weeks
	Measure1								Placebo	122	22	1	1	
6B	Baeten (29)	2015	53 centers in 13 countries	RCT	219	43.3 ± 12.9	153 (69.9)	uveitis	Secukinumab	145	21	1	0	52 weeks
	Measure2								Placebo	74	13	0	0	
7	Heijde (45)	2023	83 sites in 14 countries	RCT	332	40.4 ± 12.3	240 (72.3)	uveitis	Bimekizumab 160 mg	221	33	0	0	16 weeks
									Placebo	111	24	0	5	
8	Kivitz (46)	2018	85 centers in 19 countries	RCT	350	43.0 ± 11.8	240 (68.6)	uveitis	Secukinumab	233	44	0	0	16 weeks
									Placebo	117	27	0	0	
9	Heijde (47)	2018	84 sites in 12 countries	RCT	341	41.7 ± 11.6	276 (80.9)	AU	Ixekizumab	164	NR	0	1	16 weeks
									Adalimumab	90		0	0	
									Placebo	87		0	0	
10	Deodhar (48)	2019	106 sites in 15 countries	RCT	316	46.1 ± 12.4	253 (80.1)	AU	Ixekizumab	212	NR	2	3	16 weeks
									Placebo	104		0	0	
11	Deodhar (49)	2018	40 sites in 8 countries	RCT	208	38.8 ± 10.4	163 (78.4)	uveitis	Golimumab	105	NR	0	0	16 weeks
									Placebo	103		0	1	
12	Martín-Mola (50)	2010	14 centers in 8 countries	RCT	84	43.2 ± 10.6	66 (78.6)	uveitis	Etanercept	45	28	1	0	12 weeks
									Placebo	39		2	0	
13	Choi (11)	2020	Korea	Cohort	175	38.1 ± 16.1	123 (70.3)	uveitis	Adalimumab	62	NR	3	7	NR
									Etanercept	37		1	4	
									Golimumab	27		0	2	
									Infliximab	49		2	8	
14	Kim (51)	2016	Korea	Cohort	143	41.1 ± 13.1	97 (67.8)	uveitis	Infliximab	66	42	0	10	NR
									Adalimumab	45	33	0	7	
									Etanercept	32	19	0	6	
15	Kwon (52)	2024	Korea	Cohort	209	44.6 ± 11.6	160 (76.6)	AU	Etanercept	99	46	14	13	NR
									Adalimumab	68	36	2	6	
									Infliximab	42	23	2	7	
16	Lie (13)	2017	Swedish	Cohort	1365	43.8 ± 12.3	995 (72.9)	AU	Adalimumab	406	114	6	25	NR
									Etanercept	354	84	37	44	
									Infliximab	605	151	24	55	
17	Ahn (14)	2022	Korea	Cohort	5938	37.2 ± 13.1	4610 (77.6)	AU	Adalimumab	2477	692	44	182	NR
									Etanercept	1218	269	64	124	
									Infliximab	1214	304	25	80	
									Golimumab	1029	223	28	62	

RCT, randomized controlled trial; AU, anterior uveitis.

### 3.3 Quality assessment

**Figure 2:**
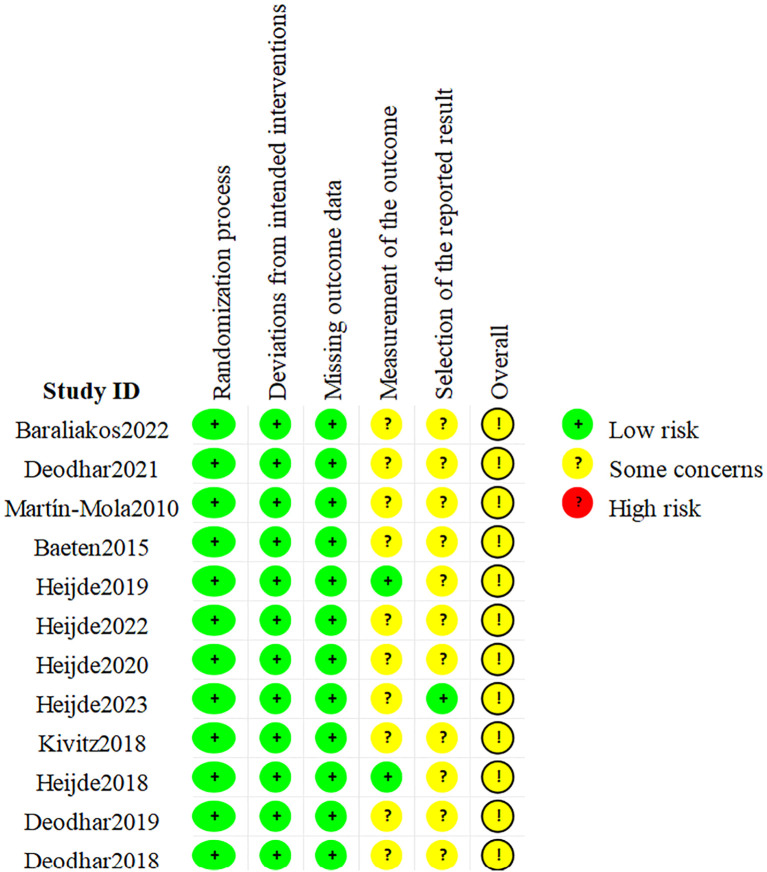
Quality assessment of RCTs via ROB 2.0.

The 12 included RCTs were assessed as having possible risks according to the ROB 2.0, as shown in **Figure 2**. Among them, all RCTs had a low risk in terms of randomization bias, established intervention bias, and missing outcome data bias, but there were some concerns in terms of unclear outcome measurement methods and/or incomplete reporting of outcome analysis protocols. Hence, the overall quality was considered some concern. In addition, the quality assessment results of the 5 cohort studies are shown in **Table 2**. The scores for cohort selection, comparability, and outcome measurement were all higher than 6 points, indicating that they were high-quality studies. Specific results are illustrated in **Figure 2** and **Table 2**.

**Table 2:** Quality assessment of cohort studies via NOS.

Study	Selection				Comparability	Outcome			Quality
	Representativeness of the exposed cohort	Selection of the non-exposed cohort	Ascertainment of exposure	Demonstration that outcome of interest was not present at start of study	Comparability of cohorts on the basis of the design or analysis	Assessment of outcome	Was follow-up long enough for outcomes to occur	Adequacy of follow up of cohorts	Scores
Choi 11	**★**	**★**	**★**	**★**	**★**	**★**	**★**	**★**	8
Kim 51	**★**	**★**	**★**	**-**	**★**	**★**	**★**	**★**	7
Kwon 52	**★**	**★**	**★**	**★**	**★**	**★**	**★**	**★**	8
Lie 13	**★**	**★**	**★**	**-**	**★**	**★**	**★**	**★**	7
Ahn 14	**★**	**★**	**★**	**★**	**★**	**★**	**★**	**★**	8

### 3.4 Network diagram of evidence

**Figure 3:**
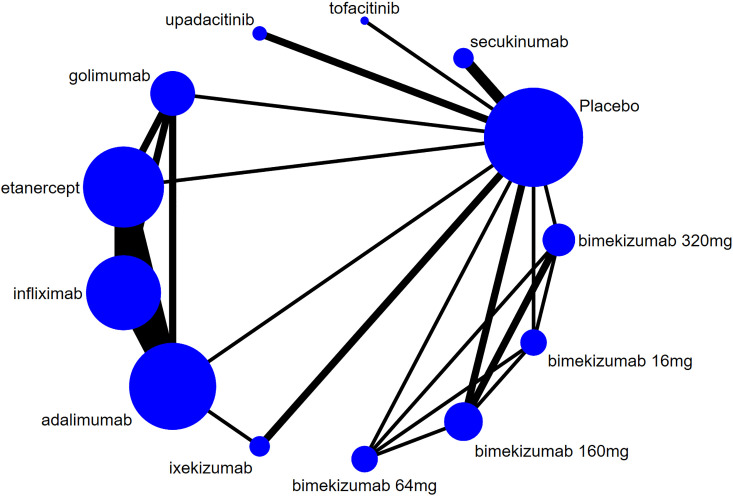
Network diagram of new-onset uveitis.

**Figure 4:**
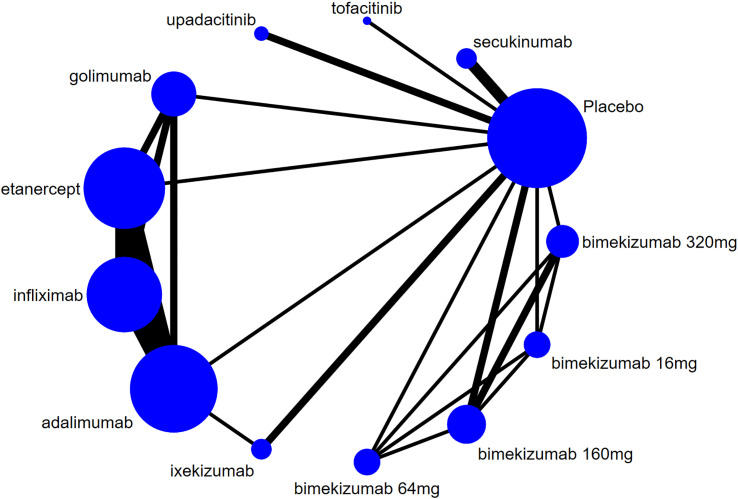
Network diagram of recurrent uveitis.

The evidence network for new-onset and recurrent uveitis is depicted in **Figures 3** and **4**, respectively, which revealed a comparative relationship between several biologics. Among them, bimekizumab formed a closed loop between different doses (16 mg, 64 mg, 160 mg, and 320 mg), and a closed loop was also observed between adalimumab, etanercept, golimumab, and infliximab. Each dot in the figure represented a biologic, the size of the dot represented the sample size of the intervention, and the thickness of lines represented the number of studies. Infliximab, adalimumab, and etanercept had the largest dots and the thickest lines, as well as closed loops.

### 3.5 New-onset uveitis

The results of league table demonstrated that adalimumab was more effective than etanercept (RR: 0.30, 95%CI: 0.22, 0.41) and golimumab (RR: 0.61, 95% CI: 0.40, 0.97) in reducing new-onset uveitis. Etanercept increased the risk of new-onset uveitis compared to golimumab (RR: 2.03, 95% CI: 1.36, 3.11) and infliximab (RR: 2.47, 95% CI: 1.81, 3.42). There were no significant differences observed with bimekizumab at any dose (320 mg, 160 mg, 64 mg, and 16 mg). Additional information is shown in **Table 3**.

**Table 3:** League table of new-onset uveitis-RR (95% CI).

Placebo												
2.69 (0.50, 15.62)	adalimumab
1.31 (0.08, 44.56)	0.49 (0.02, 24.03)	bimekizumab 16 mg
1.31 (0.08, 44.43)	0.49 (0.02, 23.54)	1.01 (0.03, 37.38)	bimekizumab 64 mg
1.33 (0.14, 10.64)	0.49 (0.03, 7.33)	1.02 (0.03, 14.71)	1.01 (0.03, 14.56)	bimekizumab 160 mg
1.24 (0.10, 16.44)	0.45 (0.02, 10.13)	0.94 (0.03, 16.75)	0.94 (0.03, 16.65)	0.93 (0.11, 8.76)	bimekizumab 320 mg
0.81 (0.15, 4.65)	0.30 (0.22, 0.41)	0.62 (0.01, 16.73)	0.61 (0.01, 16.80)	0.62 (0.04, 10.33)	0.67 (0.03, 14.69)	etanercept
1.65 (0.30, 9.65)	0.61 (0.40, 0.97)	1.25 (0.03, 34.46)	1.25 (0.03, 34.52)	1.26 (0.08, 21.07)	1.35 (0.06, 30.03)	2.03 (1.36, 3.11)	golimumab
2.02 (0.37, 11.81)	0.75 (0.52, 1.09)	1.52 (0.03, 41.91)	1.52 (0.03, 42.09)	1.54 (0.10, 25.89)	1.65 (0.07, 36.62)	2.47 (1.81, 3.42)	1.22 (0.76, 1.92)	infliximab
1.46 (0.27, 6.97)	0.53 (0.05, 5.47)	1.08 (0.02, 27.25)	1.08 (0.02, 26.91)	1.09 (0.08, 16.29)	1.17 (0.06, 23.11)	1.76 (0.16, 17.84)	0.87 (0.08, 8.82)	0.71 (0.06, 7.34)	secukinumab
0.98 (0.03, 38)	0.36 (0.01, 19.85)	0.72 (0, 71.51)	0.72 (0, 71.49)	0.75 (0.01, 51.00)	0.80 (0.01, 65.27)	1.20 (0.02, 65.41)	0.59 (0.01, 32.42)	0.48 (0.01, 26.42)	0.68 (0.01, 35.95)	tofacitinib
1.59 (0.25, 13.54)	0.60(0.05, 8.94)	1.22 (0.02, 39.90)	1.21 (0.02, 40.55)	1.23 (0.07, 25.29)	1.32 (0.05, 35.09)	1.98 (0.15, 29.15)	0.98 (0.07, 14.54)	0.80 (0.06, 12.00)	1.12 (0.10, 16.08)	1.66 (0.03, 107.09)	upadacitinib
1.20 (0.22, 5.88)	0.45 (0.04, 3.67)	0.89 (0.02, 22.80)	0.88 (0.02, 22.64)	0.90(0.06, 13.62)	0.96 (0.04, 19.53)	1.48 (0.15, 12.09)	0.73 (0.07, 6.06)	0.60 (0.06, 4.98)	0.82 (0.08, 8.23)	1.21 (0.02, 62.56)	0.73 (0.05, 8.74)	ixekizumab

**Figure 5:**
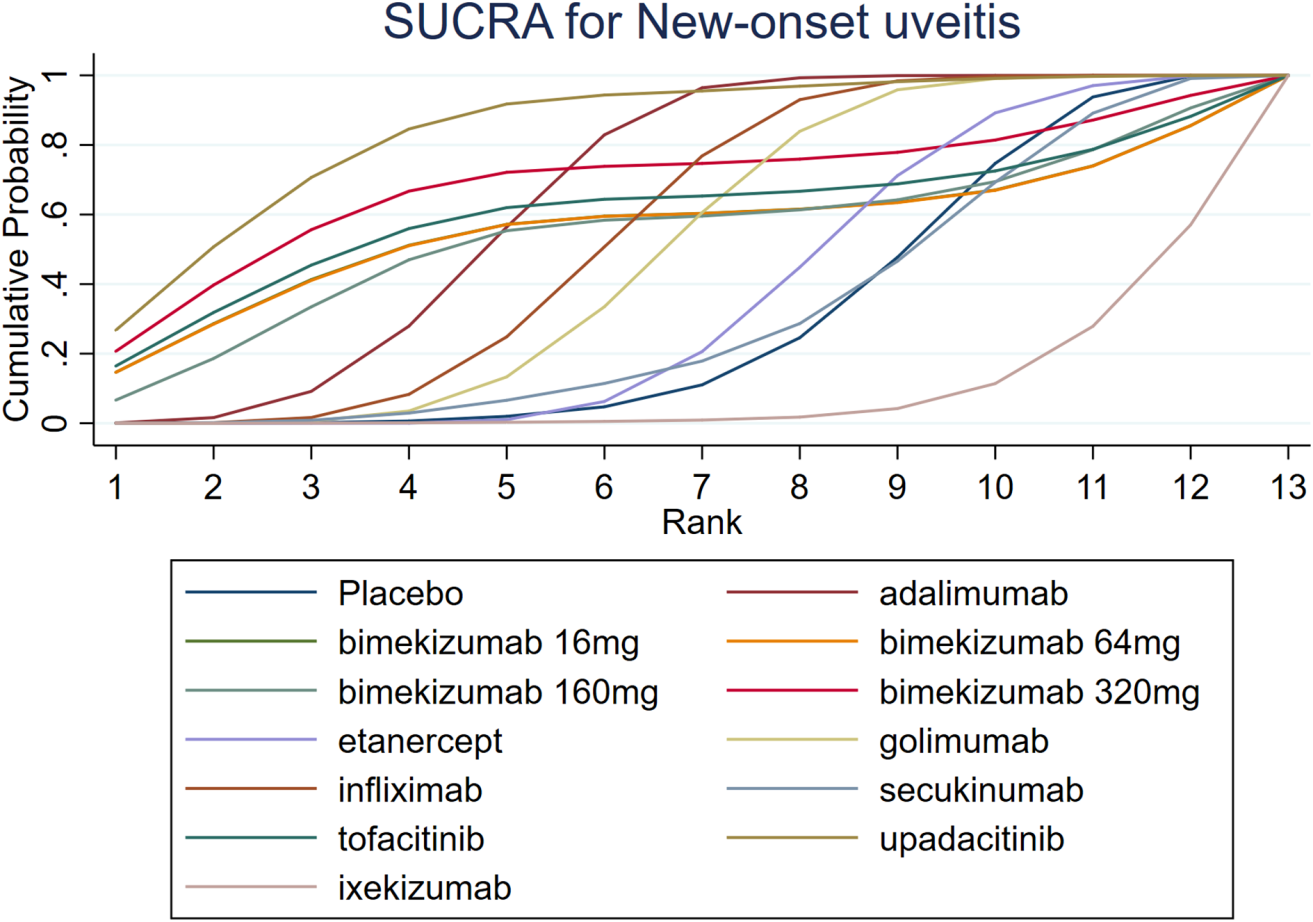
SUCRA for new-onset uveitis.

The results of SUCRA indicated that upadacitinib (84.0%) was the most efficacious, followed by bimekizumab 320 mg (68.3%) and adalimumab (64.5%). Additionally, the SUCRA of ixekizumab (8.7%) was lower than that of placebo (29.9%). Further SUCRA data are shown in **Figure 5**.

A comprehensive analysis of SUCRA and league table results revealed discrepancies. While adalimumab had a higher SUCRA ranking than infliximab among TNF-α inhibitors, the league table showed no statistically significant difference. Similarly, for JAK inhibitors, upadacitinib’s SUCRA ranking was higher than tofacitinib’s, but the league table did not demonstrate a significant difference. Among IL-17 inhibitors, bimekizumab 320 mg had a significantly higher SUCRA ranking than ixekizumab, yet the league table results again failed to show a statistically significant difference.

### 3.6 Recurrent uveitis

The results of league table indicated that adalimumab exhibited a better effect in reducing the recurrence of uveitis compared to etanercept (RR: 0.70, 95% CI: 0.58, 0.84). Compared to infliximab and golimumab, the RRs of etanercept for recurrent uveitis were 1.37 (95% CI: 1.12, 1.68) and 1.70 (95% CI: 1.30, 2.27), respectively. Compared to secukinumab, bimekizumab 160 mg was more effective in reducing recurrent uveitis (RR: 0.13, 95% CI: 0.01, 0.94), and the difference was statistically significant (P < 0.05). There were no significant differences observed with bimekizumab at any dose (320 mg, 160 mg, 64 mg, and 16 mg). More information is presented in **Table 4**.

**Table 4:** League table of recurrent uveitis-RR (95% CI).

Placebo												
1.26 (0.25, 6.60)	adalimumab											
2.35 (0.22, 73.49)	1.92 (0.10, 81.49)	bimekizumab 16mg										
2.35 (0.23, 72.15)	1.93 (0.10, 79.14)	1.00 (0.03, 38.15)	bimekizumab 64mg									
5.83 (1.46, 32.82)	4.74 (0.52, 48.39)	2.52 (0.07, 37.26)	2.53 (0.07, 37.11)	bimekizumab 160mg								
3.10 (0.37, 38.72)	2.49 (0.16, 48.06)	1.30 (0.03, 27.89)	1.30 (0.04, 28.03)	0.53 (0.05, 5.45)	bimekizumab 320mg							
0.88 (0.18, 4.59)	0.70 (0.58, 0.84)	0.36 (0.01, 6.76)	0.36 (0.01, 6.79)	0.15 (0.01, 1.33)	0.28 (0.01, 4.29)	etanercept						
1.50 (0.30, 7.85)	1.19 (0.91, 1.57)	0.62 (0.01, 11.53)	0.62 (0.01, 11.6)	0.25 (0.02, 2.27)	0.48 (0.02, 7.32)	1.70 (1.30, 2.27)	golimumab					
1.20 (0.24, 6.34)	0.96 (0.79, 1.17)	0.50 (0.01, 9.27)	0.49 (0.01, 9.32)	0.20 (0.02, 1.83)	0.38 (0.02, 5.90)	1.37 (1.12, 1.68)	0.81 (0.60, 1.07)	infliximab				
0.81 (0.16, 2.96)	0.62 (0.06, 5.13)	0.32 (0.01, 5.11)	0.32 (0.01, 5.07)	0.13 (0.01, 0.94)	0.25 (0.01, 3.22)	0.89 (0.09, 7.35)	0.52 (0.05, 4.30)	0.65 (0.07, 5.37)	secukinumab			
3.78 (0.41, 115.08)	3.12 (0.18, 127.67)	1.62 (0.03, 97.98)	1.63 (0.03, 95.92)	0.65 (0.04, 24.75)	1.26 (0.04, 64.3)	4.47 (0.27, 182.68)	2.61 (0.15, 107.31)	3.26 (0.19, 133.92)	5.00 (0.35, 199.04)	tofacitinib		
1.60 (0.25, 13.77)	1.28 (0.11, 18.70)	0.66 (0.01, 16.50)	0.67 (0.01, 16.21)	0.27 (0.02, 3.49)	0.51 (0.02, 10.53)	1.83 (0.15, 26.85)	1.07 (0.09, 15.72)	1.33 (0.11, 19.57)	2.06 (0.20, 28.13)	0.41 (0.01, 9.19)	upadacitinib	
0.86 (0.15, 3.78)	0.68 (0.08, 4.78)	0.35 (0.01, 6.04)	0.35 (0.01, 6.09)	0.14 (0.01, 1.13)	0.27 (0.01, 3.78)	0.97 (0.11, 6.88)	0.57 (0.07, 4.06)	0.71 (0.08, 5.04)	1.08 (0.13, 9.23)	0.21 (0.01, 3.33)	0.52 (0.03, 5.83)	ixekizumab

**Figure 6:**
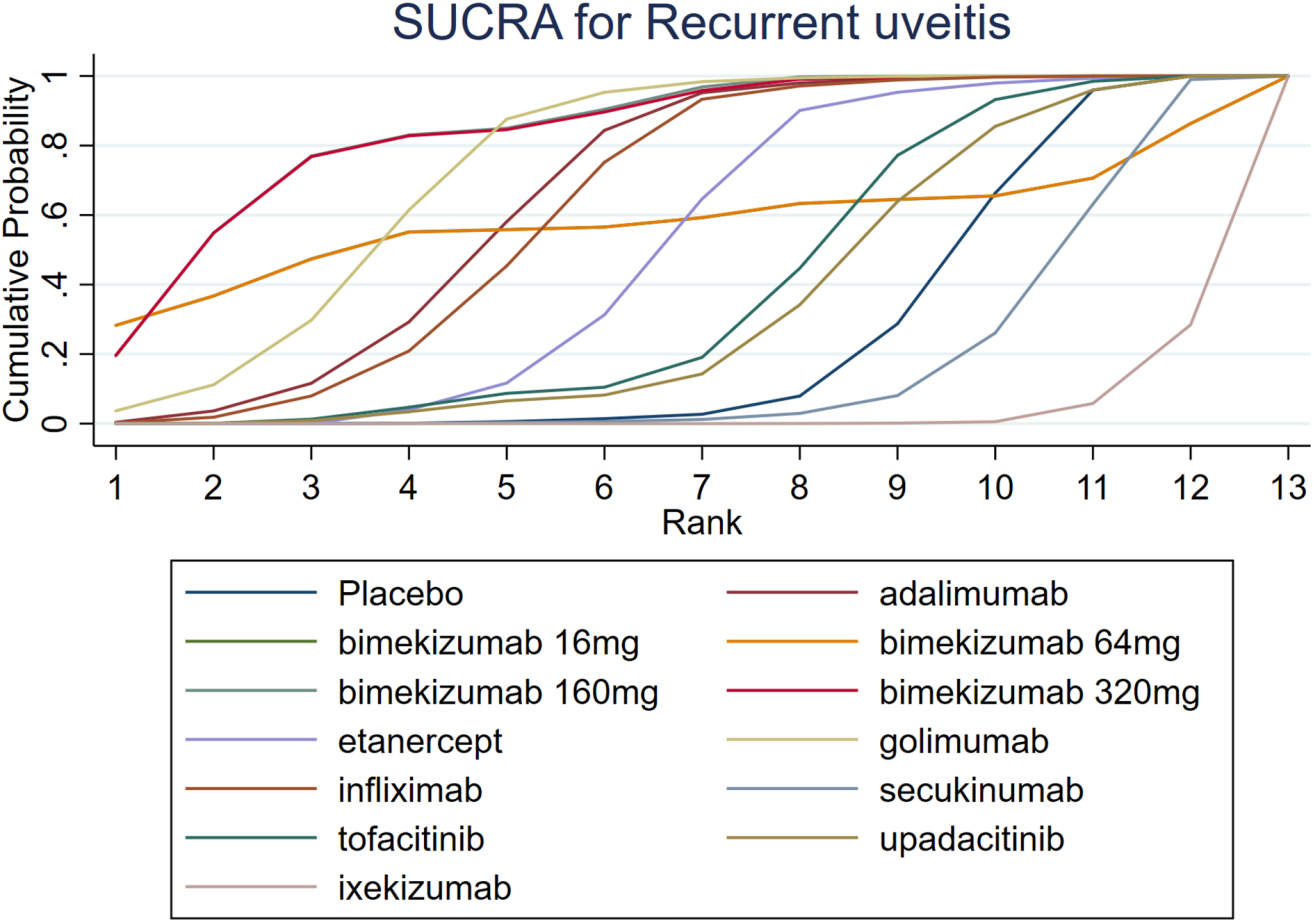
SUCRA for recurrent uveitis.

The results of SUCRA indicated that bimekizumab 160 mg (83.9%) was the most effective in reducing recurrent uveitis, followed by bimekizumab 320 mg (83.5%) and golimumab (73.9%). Besides, the SUCRA ixekizumab (2.9%) was lower than that of secukinumab (16.8%) and placebo (25.3%). Details of SUCRA are presented in **Figure 6**.

A comprehensive analysis of SUCRA and league table results revealed discrepancies. While golimumab had a higher SUCRA ranking than adalimumab among TNF-α inhibitors, the league table showed no statistically significant difference. Similarly, for JAK inhibitors, upadacitinib’s SUCRA ranking was lower than tofacitinib’s, but the league table did not demonstrate a significant difference. Among IL-17 inhibitors, bimekizumab 160 mg had a significantly higher SUCRA ranking than ixekizumab, yet the league table results again failed to show a statistically significant difference.

### 3.7 Combined cumulative probability of new-onset and recurrent uveitis

**Figure 7:**
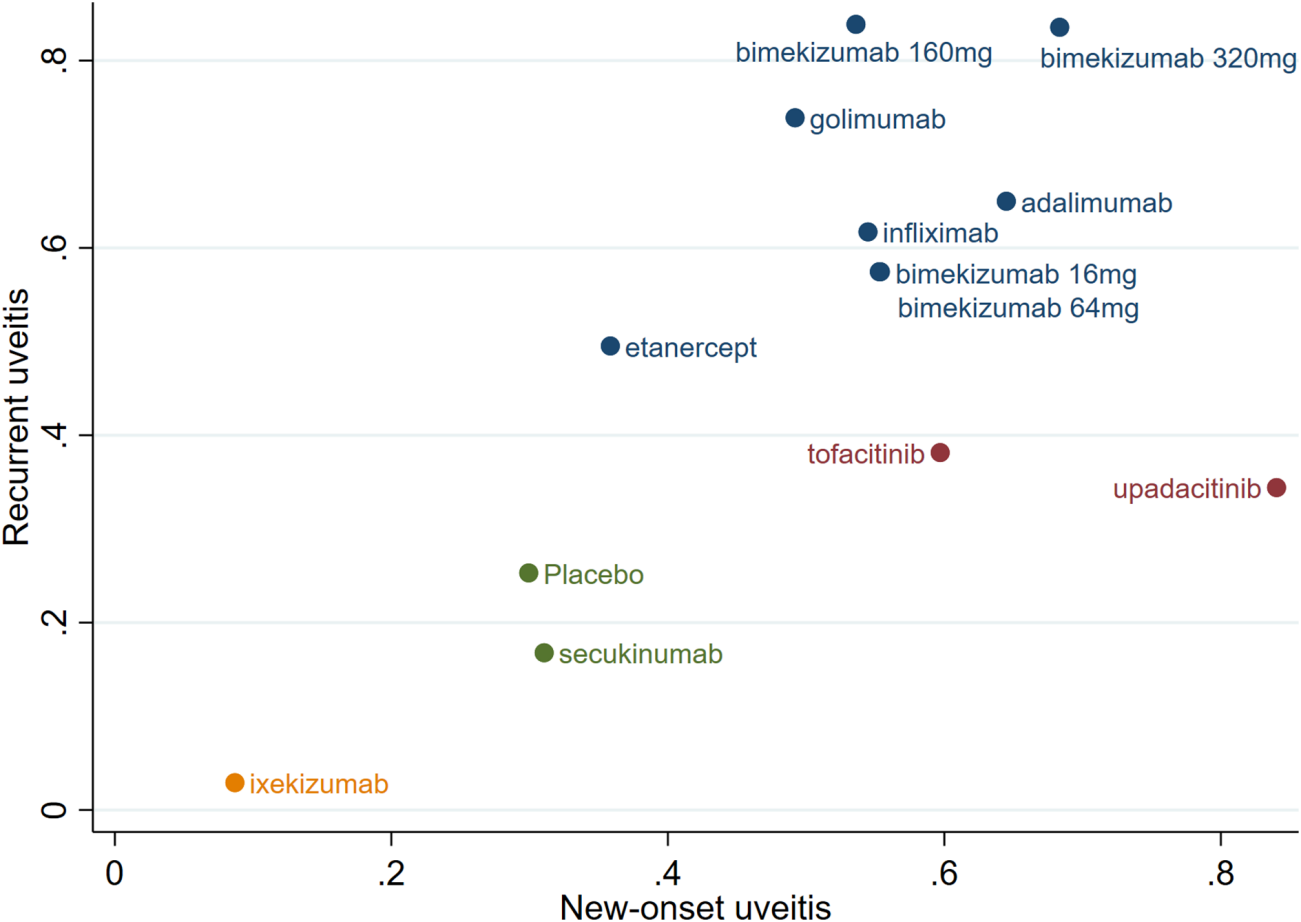
Combined cumulative probability of new-onset and recurrent uveitis.

According to the SUCRA for new-onset and recurrent uveitis, significant differences in efficacy among the drugs were observed, as shown in **Figure 7**. Bimekizumab 320/160 mg, adalimumab and golimumab demonstrated significant advantages in treating uveitis. Among them, bimekizumab 320 mg exhibited the best efficacy; etanercept, infliximab, bimekizumab 16/64 mg, tofacitinib, and upadacitinib showed moderate efficacy; ixekizumab and secukinumab exhibited lower efficacy than placebo and had limited effects in reducing the incidence of uveitis.

### 3.8 Publication bias

**Figure 8:**
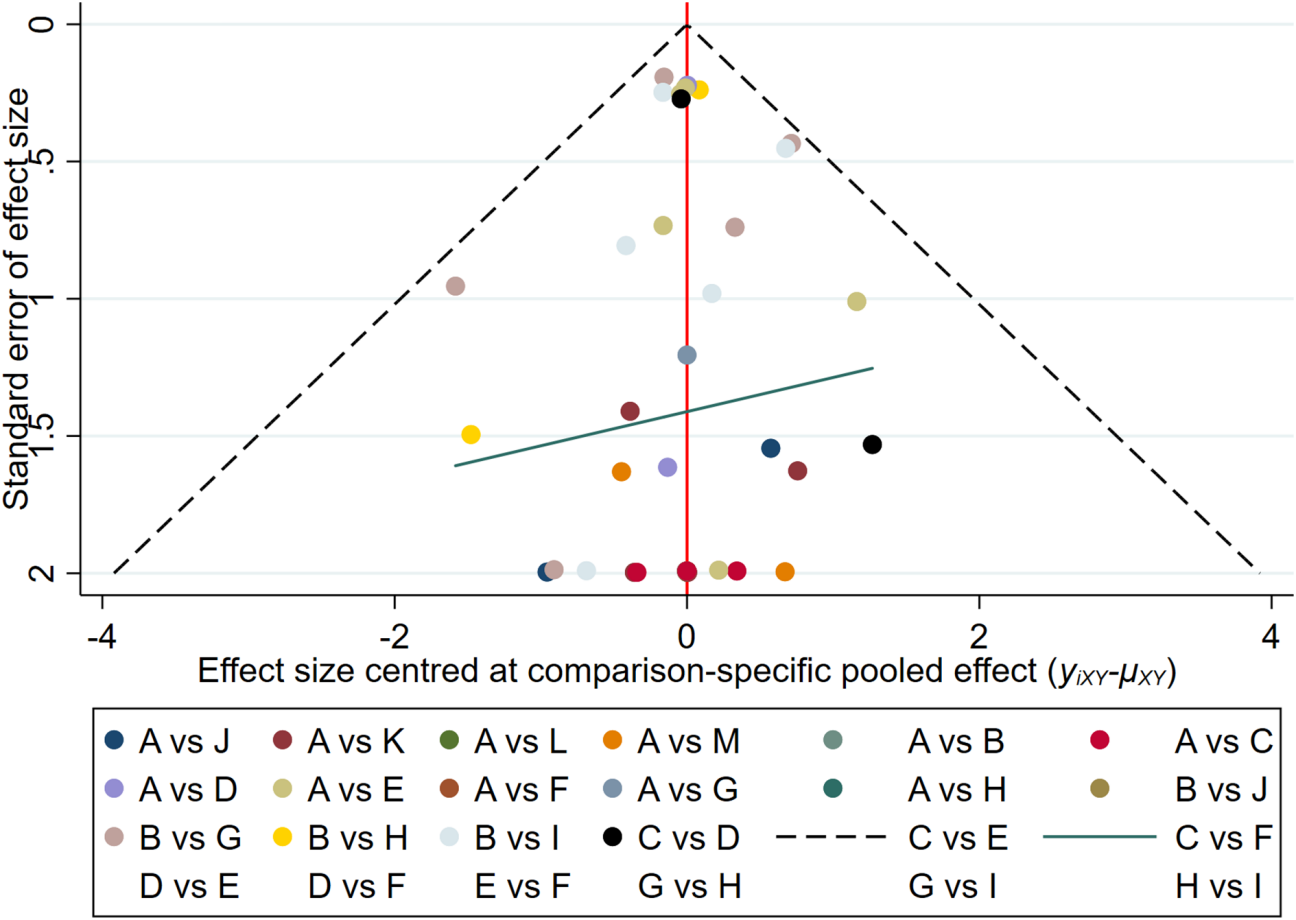
Funnel plot of new-onset uveitis.

**Figure 9:**
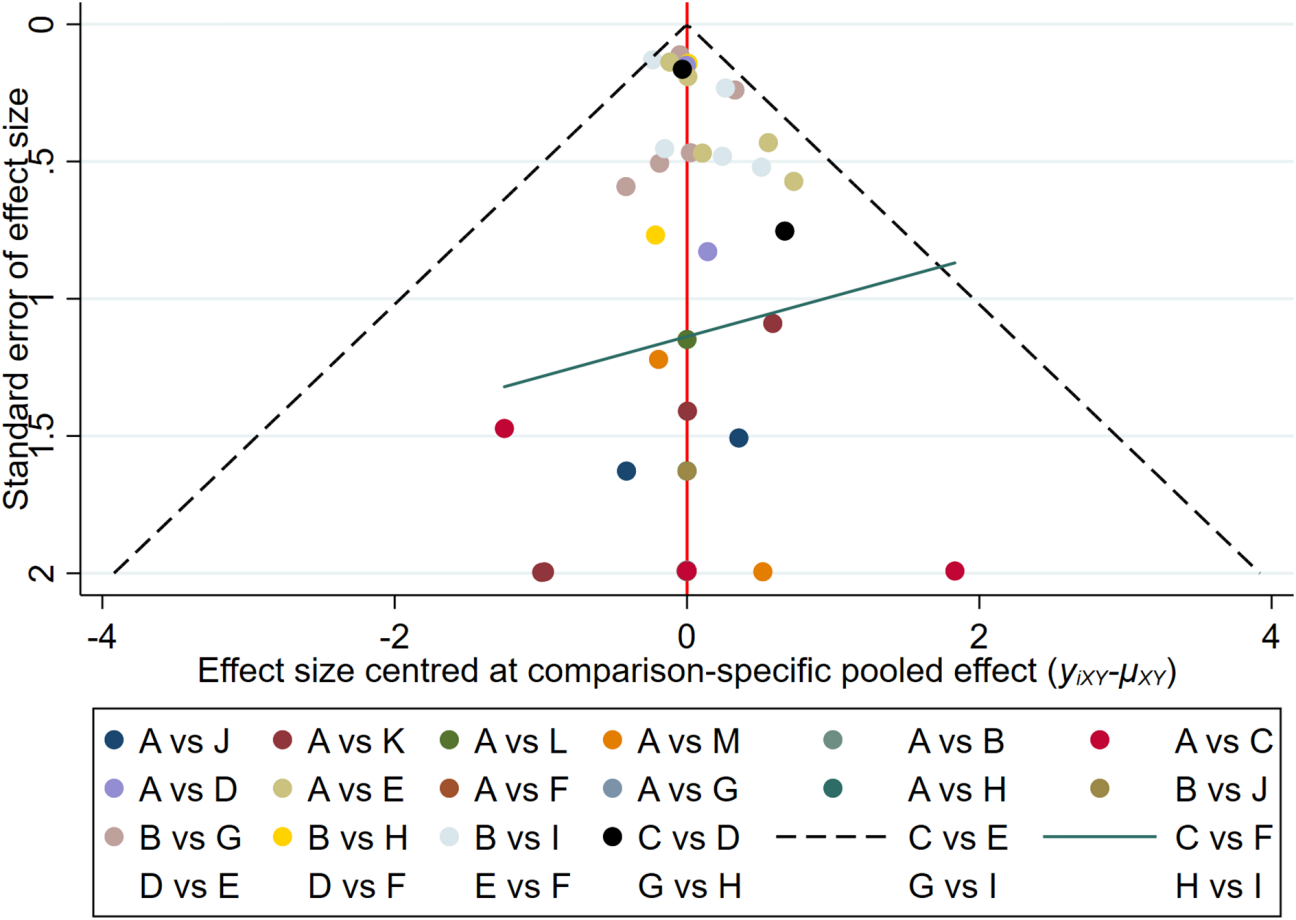
Funnel plot of recurrent uveitis.

The results of the funnel plot indicated that the studies on new-onset and recurrent uveitis were generally symmetrical between left and right, and there was a low likelihood of publication bias. Details are presented in **Figures 8** and **9**.

### 3.9 Heterogeneity and consistency test

The heterogeneity test results showed that the I² values for all studies on new-onset and recurrent uveitis were below 30%, indicating low heterogeneity among the included studies. Details are presented in Attachment 3; The consistency test results showed that the tests for new-onset uveitis and recurrent uveitis yielded χ²(7) = 5.35 (P = 0.618) and χ²(7) = 5.17 (P = 0.639), respectively, neither of which reached statistical significance (P > 0.05). Detailed results can be found in Attachment 4.

## 4 Discussion

The current study comprehensively included three types of biologics for the first time, i.e., TNF-α inhibitors, IL-17 inhibitors, and JAK inhibitors, involving 9 drugs (adalimumab, etanercept, golimumab, infliximab, secukinumab, bimekizumab, ixekizumab, tofacitinib, and upadacitinib) to analyze the impact of these drugs on the risk of new and recurrent uveitis in AS patients. Utilizing an NMA, this study not only analyzed and compared all available direct and indirect evidence for different treatments, but also compensated for the shortcomings of head-to-head comparisons in traditional MAs (19). In addition, the division of uveitis into two outcome indicators (new-onset and recurrence) was more comprehensive and reasonable.

This NMA indicated that upadacitinib, bimekizumab 320 mg and adalimumab were excellent in reducing the risk of new-onset uveitis, and bimekizumab 160 mg, bimekizumab 320 mg and golimumab were excellent in reducing the risk of recurrent uveitis. Overall, bimekizumab 320 mg, bimekizumab 160 mg, and adalimumab performed best in reducing the risk of new-onset and recurrent uveitis, whereas ixekizumab was superior to placebo in reducing the new-onset, recurrence, and combined risks.

Uveitis is an immune-mediated inflammatory disease, and its pathogenesis is mainly linked to the abnormal activation of Th1 and Th17 helper T cells. These activated T cells secrete several inflammatory cytokines, such as TNF-α, IL-17, IL-23 and IL-6, which in turn trigger an inflammatory response in the eye (20, 21). TNF-α plays a pivotal role in the pathogenesis of immune-inflammatory diseases, and TNF-α inhibitors have been widely applied in the clinical treatment of autoimmune diseases. Previous studies have shown that etanercept, a soluble TNF receptor fusion protein, is less effective in preventing AU than TNF mAbs adalimumab and infliximab (13, 22–24). The current study also reached a consistent conclusion that etanercept increased the risk of both new-onset and recurrent uveitis compared to adalimumab, golimumab, and infliximab. This clinical difference may be closely related to the mechanisms of action of the two drug classes and the characteristics of the ocular immune microenvironment. TNF-α exists in two forms: soluble (sTNF-α) and transmembrane (mTNF-α), which mediate distinct biological effects by binding to TNFR1 and TNFR2, respectively. sTNF-α primarily transmits pro-inflammatory signals via TNFR1, while mTNF-α regulates immune balance and tissue repair through TNFR2 (25). Etanercept, a fusion protein composed of the extracellular domain of TNFR2 and the IgG1 Fc segment, selectively neutralizes sTNF-α and inhibits the TNFR1 pathway. Critically, it does not bind to the membrane-bound mTNF-α or induce apoptosis. Furthermore, its larger molecular weight results in significantly lower ocular permeability compared to mAbs, making it difficult to achieve effective therapeutic concentrations in uveal tissue (26, 27). Conversely, mAbs such as adalimumab and infliximab target both sTNF-α and mTNF-α, thereby blocking both TNFR1 and TNFR2 signaling pathways. Specifically, the anti-inflammatory effect of TNFR2 is crucial for regulating ocular immunity and effectively managing ocular inflammation (28). In summary, the observed differences in uveitis prevention between etanercept and monoclonal TNF-α inhibitors likely arise from their selective binding to sTNF-α/mTNF-α, their differential blockade of receptor pathways, and variations in ocular drug distribution.

For IL-17 inhibitors, the abnormal activation of Th17 cells is a key factor in a variety of inflammatory and autoimmune diseases. IL-17A and IL-17F play a pivotal role in mediating the inflammatory response by driving tissue inflammation through the induction of pro-inflammatory cytokines and chemokines. Secukinumab and ixekizumab are high-affinity IL-17A mAbs that have been shown to significantly reduce the signs and symptoms of AS (29, 30). However, this study found that these agents were associated with an increased risk of uveitis onset or recurrence in AS patients. Conversely, bimekizumab, which dually targets both IL-17A and IL-17F, has been shown to more effectively suppress cytokine responses and neutrophil chemotaxis *in vitro*, exhibiting stronger anti-inflammatory effects. In this study, bimekizumab demonstrated a lower association with uveitis (31). A study of autoimmune uveitis and its animal model (EAU) demonstrated that blocking IL-17A alone in autopathogenic Th17 cells did not reduce their pathogenicity, but increased the expression of GM-CSF and IL-17F. Blocking IL-17F alone did not cause upregulation of IL-17A and GM-CSF in Th17 cells (32). Therefore, the limitations of Secukinumab and Ixekizumab in preventing uveitis may be related to their inability to block the compensatory effects of IL-17F. In contrast, Bimekizumab, by simultaneously targeting both IL-17A and IL-17F, demonstrates superior uveitis prevention in clinical trials. This difference may be associated with the independent role of IL-17F in the pathogenesis of uveitis.

JAK inhibitors (JAKnibs) bind to JAK phosphorylation sites in cells to block the signal transduction of various cytokine receptors, show potent immunosuppressive activity, and are a promising treatment for autoimmune diseases (33, 34). Studies indicated that AS patients had higher expression of JAK-1 and JAK-3 (35). Dysregulation of the JAK-STAT signaling pathway can lead to a range of autoimmune diseases, including inflammatory bowel disease, multiple sclerosis, psoriasis, and uveitis (36). Tofacitinib is primarily a JAK1/3 inhibitor, which is ineffective in inhibiting JAK2 activity (37). Upadacitinib is a reversible and selective JAK1 inhibitor (38). The results of the current study revealed that tofacitinib and upadacitinib were more effective in managing new-onset uveitis than in recurrent cases. The reason may be that tofacitinib and upadacitinib can penetrate the blood-retinal barrier more effectively due to their small molecular weight. They are more effective in controlling the initial mild inflammation in new-onset uveitis. However, in recurrent uveitis, drug penetration may be reduced, and efficacy is limited due to the multiple repair of ocular tissues resulting in structural changes such as fibrosis, scarring, and angiogenesis (39). Therefore, JAK inhibitors are a better choice for new-onset uveitis than for recurrent uveitis.

The NMA of the effects of different biologics on new-onset and recurrent uveitis in AS patients is clinically important. First, uveitis is a common ocular complication in AS patients, often leading to visual impairment and decreased quality of life. A systematic review and NMA can comprehensively evaluate the efficacy and safety of different biologics in preventing new-onset uveitis or reducing recurrent uveitis, thereby providing clinicians with a clearer basis for choosing treatment options. Furthermore, such analyses can reveal potential differences between different biologics in the prevention of uveitis, which may help to optimize individualized treatment and improve long-term prognosis for patients. This provides a reliable evidence base for the clinical development of comprehensive programs to manage patients with AS combined with uveitis.

This study has several limitations. (1) While this study investigated the impact of nine biologics on the risk of new-onset and recurrent uveitis in AS patients, the number of RCTs for some biologics was limited. To supplement the sample size and expand the external applicability of the findings, cohort studies were also incorporated. However, RCTs and cohort studies differ inherently in intervention standardization, control of confounders, and risk of follow-up bias. Despite the confirmation of result stability through heterogeneity and consistency tests, the combination of these two types of studies may have reduced statistical power, thus potentially affecting the comprehensive assessment of the overall effect. (2) The primary conclusions of this study were derived from SUCRA rankings, supplemented by league table results. Although heterogeneity and consistency tests indicated stable results, the small sample size for certain biologics could have led to SUCRA rankings being unduly influenced by limited data, which might affect their reliability. Furthermore, the low frequency of some outcome events in the league table’s pairwise comparisons resulted in wide confidence intervals with considerable overlap, affecting the precision of the results. Therefore, future studies should increase sample sizes and systematically interpret SUCRA rankings and league table results in conjunction with clinical realities, such as the accessibility and safety of interventions. This would enhance the robustness and clinical applicability of the conclusions. (3) The analysis of recurrent uveitis risk was constrained by data availability. Although some included studies reported a history of prior uveitis, most did not provide this information or stratify by specific interventions. Despite efforts to contact the authors of the original studies to obtain relevant data, some data remained unavailable, potentially leading to an underestimation of the true incidence of recurrent uveitis and a failure to accurately adjust for the confounding effects of baseline disease history. This could have led to an overestimation of the therapeutic effects of the interventions. (4) Subgroup analyses were limited, particularly concerning the exploration of regional and ethnic variations, uveitis subtypes (e.g., AU, intermediate uveitis, posterior uveitis, and panuveitis), and the dose-response relationship of biologics. Specifically, while the study involved different doses of bimekizumab (16 mg, 64 mg, 160 mg, 320 mg), the small sample sizes within each dose group and inconsistent follow-up times precluded a detailed analysis of dose-related differences in uveitis risk. Future research should address the following directions: First, dynamic stratified studies should be conducted, developing differentiated research protocols for primary prevention in populations without a history of uveitis and for relapse prevention in those with a history. Careful stratification of baseline characteristics is essential to clarify the effectiveness of interventions in different risk populations. Second, outcome measures should be standardized by adopting internationally recognized criteria (e.g., the SUN Working Group criteria) for defining uveitis events. The dimensions of efficacy assessment (e.g., flare frequency, duration, and visual recovery) should be refined to enhance comparability across studies. Third, the dose-response relationship of biologics should be investigated by establishing different dose groups and monitoring drug concentrations to optimize individualized treatment regimens, thereby balancing efficacy and safety. Fourth, mechanistic research should be strengthened to elucidate the specific mechanisms by which biologics modulate immune-inflammatory pathways (e.g., TNF-α, IL-6, IL-17 signaling pathways). This will provide scientific evidence for the development of precision-targeted therapeutic strategies.

## 5 Conclusion

In conclusion, the efficacy of JAK inhibitors for new-onset uveitis was superior to that for recurrent cases in AS patients. Inhibition of IL-17A alone might increase the risk of new-onset or recurrent uveitis, but simultaneous inhibition of IL-17A and IL-17F significantly reduced the risk of uveitis. Among TNF-α inhibitors, etanercept was linked to a higher risk of new-onset and recurrent uveitis in AS patients than other similar biologics. Therefore, clinicians may individualize and precisely select drugs for the prevention and treatment of uveitis in AS patients.
