# Supplementary material for: Risk of new-onset and recurrent uveitis with different biologics for ankylosing spondylitis: a network meta-analysis
Source: Front Immunol. 2025 Jun 20;16:1556313. doi: 10.3389/fimmu.2025.1556313 (PMC12226306; doi:10.3389/fimmu.2025.1556313)
Supplement: Supplementary file 2 [file Table2.docx]

Retrieval strategy

| Pubmed | | |
| --- | --- | --- |
| # | Query | Results |
| #20 | #3 AND #19 | 3,715 |
| #19 | #4 OR #5 OR #6 OR #7 OR #8 OR #9 OR #10 OR #11 OR #12 OR #13 OR #14 OR #15 OR #16 OR #17 OR #18 | 89,212 |
| #18 | "abrilada"[Title/Abstract] OR "adalimumab"[Title/Abstract] OR "adaly"[Title/Abstract] OR "altebrel"[Title/Abstract] OR "amgevita"[Title/Abstract] OR "amjevita"[Title/Abstract] OR "amsparity"[Title/Abstract] OR "anti TNF"[Title/Abstract] OR "anti tumo*r necrosis factor agent"[Title/Abstract] OR "avakine"[Title/Abstract] OR "avent"[Title/Abstract] OR "avsola"[Title/Abstract] OR "benepali"[Title/Abstract] OR "bimekizumab"[Title/Abstract] OR "bimzelx"[Title/Abstract] OR "brenzys"[Title/Abstract] OR "certolizumab pegol"[Title/Abstract] OR "cimzia"[Title/Abstract] OR "cinnora"[Title/Abstract] OR "cosentyx"[Title/Abstract] OR "CTLA 8"[Title/Abstract] OR "CTLA8"[Title/Abstract] OR "cyltezo"[Title/Abstract] OR "Cytokine CX2"[Title/Abstract] OR "Cytokine ML 1"[Title/Abstract] OR "cytotoxic T lymphocyte antigen 8"[Title/Abstract] OR "cytotoxic T lymphocyte protein 8"[Title/Abstract] OR "D2E7 Antibody"[Title/Abstract] OR "davictrel"[Title/Abstract] OR "embrel"[Title/Abstract] OR "enbrel"[Title/Abstract] OR "enerceptan"[Title/Abstract] OR "erelzi"[Title/Abstract] OR "etacept"[Title/Abstract] OR "etanar"[Title/Abstract] OR "etanercept"[Title/Abstract] OR "eticovo"[Title/Abstract] OR "exemptia"[Title/Abstract] OR "filgotinib"[Title/Abstract] OR "flammegis"[Title/Abstract] OR "flixabi"[Title/Abstract] OR "fyzoclad"[Title/Abstract] OR "golimumab"[Title/Abstract] OR "hadlima"[Title/Abstract] OR "halimatoz"[Title/Abstract] OR "hefiya"[Title/Abstract] OR "hukyndra"[Title/Abstract] OR "hulio"[Title/Abstract] OR "humira"[Title/Abstract] OR "hyrimoz"[Title/Abstract] OR "idacio"[Title/Abstract] OR "IL 17*"[Title/Abstract] OR "imraldi"[Title/Abstract] OR "infimab"[Title/Abstract] OR "infinitam"[Title/Abstract] OR "inflectra"[Title/Abstract] OR "infliximab"[Title/Abstract] OR "interleukin 17*"[Title/Abstract] OR "Interleukin 25"[Title/Abstract] OR "ixekizumab"[Title/Abstract] OR "ixifi"[Title/Abstract] OR "JAK inhibitor*"[Title/Abstract] OR "Janus kinase inhibitor*"[Title/Abstract] OR "jaquinus"[Title/Abstract] OR "jyseleca"[Title/Abstract] OR "kromeya"[Title/Abstract] OR "libmyris"[Title/Abstract] OR "lifmior"[Title/Abstract] OR "MAb cA2"[Title/Abstract] OR "mabura"[Title/Abstract] OR "Monoclonal Antibody cA2"[Title/Abstract] OR "monoclonal antibody D2E7"[Title/Abstract] OR "nepexto"[Title/Abstract] OR "opinercept"[Title/Abstract] OR "pegylated tumo*r necrosis factor alpha antibody Fab fragment"[Title/Abstract] OR "qletli"[Title/Abstract] OR "raheara"[Title/Abstract] OR "remicade"[Title/Abstract] OR "remsima"[Title/Abstract] OR "renflexis"[Title/Abstract] OR "reumatocept"[Title/Abstract] OR "revellex"[Title/Abstract] OR "rinvoq"[Title/Abstract] OR "scapho"[Title/Abstract] OR "secukinumab"[Title/Abstract] OR "shinponi"[Title/Abstract] OR "simponi"[Title/Abstract] OR "simziya"[Title/Abstract] OR "solymbic"[Title/Abstract] OR "sulinno"[Title/Abstract] OR "taltz"[Title/Abstract] OR "tasocitinib"[Title/Abstract] OR "TNF a"[Title/Abstract] OR "TNF Antagonist*"[Title/Abstract] OR "TNF Blocker*"[Title/Abstract] OR "TNF inhibitor*"[Title/Abstract] OR "TNF Receptor Type II IgG Fusion Protein"[Title/Abstract] OR "TNFR Fc Fusion Protein"[Title/Abstract] OR "TNT Receptor Fusion Protein"[Title/Abstract] OR "TNTR Fc"[Title/Abstract] OR "tofacitinib"[Title/Abstract] OR "trudexa"[Title/Abstract] OR "tumo*r necrosis factor inhibitor*"[Title/Abstract] OR "tumo*r necrosis factor receptor Fc fusion protein"[Title/Abstract] OR "tumor necrosis factor alpha inhibitor"[Title/Abstract] OR "Tumor Necrosis Factor Antagonist"[Title/Abstract] OR "Tumor Necrosis Factor Blocker*"[Title/Abstract] OR "tunex"[Title/Abstract] OR "upadacitinib"[Title/Abstract] OR "xcimzane"[Title/Abstract] OR "xeljanz"[Title/Abstract] OR "yisaipu"[Title/Abstract] OR "yuflyma"[Title/Abstract] OR "yusimry"[Title/Abstract] OR "zessly"[Title/Abstract] OR "zymfentra"[Title/Abstract] | 83,952 |
| #17 | GLPG0634 [Supplementary Concept] | 131 |
| #16 | upadacitinib [Supplementary Concept] | 400 |
| #15 | tofacitinib [Supplementary Concept] | 1,737 |
| #14 | ixekizumab [Supplementary Concept] | 532 |
| #13 | golimumab [Supplementary Concept] | 823 |
| #12 | secukinumab [Supplementary Concept] | 1,148 |
| #11 | bimekizumab [Supplementary Concept] | 87 |
| #10 | Janus Kinase Inhibitors[MeSH Terms] | 2,004 |
| #9 | Interleukin-17[MeSH Terms] | 15,592 |
| #8 | Tumor Necrosis Factor Inhibitors[MeSH Terms] | 2,919 |
| #7 | Certolizumab Pegol[MeSH Terms] | 762 |
| #6 | Infliximab[MeSH Terms] | 12,441 |
| #5 | Adalimumab[MeSH Terms] | 7,296 |
| #4 | Etanercept[MeSH Terms] | 6,624 |
| #3 | #1 OR #2 | 22,949 |
| #2 | "ankyl* spond*litis"[Title/Abstract] OR "ankylo* spondyl*arthr*"[Title/Abstract] OR "ankylosing spine"[Title/Abstract] OR "Be*hterew* disease"[Title/Abstract] OR "bekhterev disease"[Title/Abstract] OR "Marie Struempell Disease"[Title/Abstract] OR "morbus bechterew"[Title/Abstract] OR "Rheumatoid Spondylitis"[Title/Abstract] OR "spin* ankylosis"[Title/Abstract] OR "spondyl* ankylopo*etica"[Title/Abstract] OR "spondylarthr*is ankylo*"[Title/Abstract] OR "vertebral ankylosis"[Title/Abstract] | 18,388 |
| #1 | Spondylitis, Ankylosing[MeSH Terms] | 16,995 |

| Embase | | |
| --- | --- | --- |
| # | Query | Results |

| #20 | #3 AND #19 | 11952 |
| --- | --- | --- |
| #19 | #4 OR #5 OR #6 OR #7 OR #8 OR #9 OR #10 OR #11 OR #12 OR #13 OR #14 OR #15 OR #16 OR #17 OR #18 | 275192 |
| #18 | 'abrilada':ab,ti,kw OR 'adalimumab':ab,ti,kw OR 'adaly':ab,ti,kw OR 'altebrel':ab,ti,kw OR 'amgevita':ab,ti,kw OR 'amjevita':ab,ti,kw OR 'amsparity':ab,ti,kw OR 'anti tnf':ab,ti,kw OR 'anti tumo*r necrosis factor agent':ab,ti,kw OR 'avakine':ab,ti,kw OR 'avent':ab,ti,kw OR 'avsola':ab,ti,kw OR 'benepali':ab,ti,kw OR 'bimekizumab':ab,ti,kw OR 'bimzelx':ab,ti,kw OR 'brenzys':ab,ti,kw OR 'certolizumab pegol':ab,ti,kw OR 'cimzia':ab,ti,kw OR 'cinnora':ab,ti,kw OR 'cosentyx':ab,ti,kw OR 'ctla 8':ab,ti,kw OR 'ctla8':ab,ti,kw OR 'cyltezo':ab,ti,kw OR 'cytokine cx2':ab,ti,kw OR 'cytokine ml 1':ab,ti,kw OR 'cytotoxic t lymphocyte antigen 8':ab,ti,kw OR 'cytotoxic t lymphocyte protein 8':ab,ti,kw OR 'd2e7 antibody':ab,ti,kw OR 'davictrel':ab,ti,kw OR 'embrel':ab,ti,kw OR 'enbrel':ab,ti,kw OR 'enerceptan':ab,ti,kw OR 'erelzi':ab,ti,kw OR 'etacept':ab,ti,kw OR 'etanar':ab,ti,kw OR 'etanercept':ab,ti,kw OR 'eticovo':ab,ti,kw OR 'exemptia':ab,ti,kw OR 'filgotinib':ab,ti,kw OR 'flammegis':ab,ti,kw OR 'flixabi':ab,ti,kw OR 'fyzoclad':ab,ti,kw OR 'golimumab':ab,ti,kw OR 'hadlima':ab,ti,kw OR 'halimatoz':ab,ti,kw OR 'hefiya':ab,ti,kw OR 'hukyndra':ab,ti,kw OR 'hulio':ab,ti,kw OR 'humira':ab,ti,kw OR 'hyrimoz':ab,ti,kw OR 'idacio':ab,ti,kw OR 'il 17*':ab,ti,kw OR 'imraldi':ab,ti,kw OR 'infimab':ab,ti,kw OR 'infinitam':ab,ti,kw OR 'inflectra':ab,ti,kw OR 'infliximab':ab,ti,kw OR 'interleukin 17*':ab,ti,kw OR 'interleukin 25':ab,ti,kw OR 'ixekizumab':ab,ti,kw OR 'ixifi':ab,ti,kw OR 'jak inhibitor*':ab,ti,kw OR 'janus kinase inhibitor*':ab,ti,kw OR 'jaquinus':ab,ti,kw OR 'jyseleca':ab,ti,kw OR 'kromeya':ab,ti,kw OR 'libmyris':ab,ti,kw OR 'lifmior':ab,ti,kw OR 'mab ca2':ab,ti,kw OR 'mabura':ab,ti,kw OR 'monoclonal antibody ca2':ab,ti,kw OR 'monoclonal antibody d2e7':ab,ti,kw OR 'nepexto':ab,ti,kw OR 'opinercept':ab,ti,kw OR 'pegylated tumo*r necrosis factor alpha antibody fab fragment':ab,ti,kw OR 'qletli':ab,ti,kw OR 'raheara':ab,ti,kw OR 'remicade':ab,ti,kw OR 'remsima':ab,ti,kw OR 'renflexis':ab,ti,kw OR 'reumatocept':ab,ti,kw OR 'revellex':ab,ti,kw OR 'rinvoq':ab,ti,kw OR 'scapho':ab,ti,kw OR 'secukinumab':ab,ti,kw OR 'shinponi':ab,ti,kw OR 'simponi':ab,ti,kw OR 'simziya':ab,ti,kw OR 'solymbic':ab,ti,kw OR 'sulinno':ab,ti,kw OR 'taltz':ab,ti,kw OR 'tasocitinib':ab,ti,kw OR 'tnf a':ab,ti,kw OR 'tnf antagonist*':ab,ti,kw OR 'tnf blocker*':ab,ti,kw OR 'tnf inhibitor*':ab,ti,kw OR 'tnf receptor type ii igg fusion protein':ab,ti,kw OR 'tnfr fc fusion protein':ab,ti,kw OR 'tnt receptor fusion protein':ab,ti,kw OR 'tntr fc':ab,ti,kw OR 'tofacitinib':ab,ti,kw OR 'trudexa':ab,ti,kw OR 'tumo*r necrosis factor inhibitor*':ab,ti,kw OR 'tumo*r necrosis factor receptor fc fusion protein':ab,ti,kw OR 'tumor necrosis factor alpha inhibitor':ab,ti,kw OR 'tumor necrosis factor antagonist':ab,ti,kw OR 'tumor necrosis factor blocker*':ab,ti,kw OR 'tunex':ab,ti,kw OR 'upadacitinib':ab,ti,kw OR 'xcimzane':ab,ti,kw OR 'xeljanz':ab,ti,kw OR 'yisaipu':ab,ti,kw OR 'yuflyma':ab,ti,kw OR 'yusimry':ab,ti,kw OR 'zessly':ab,ti,kw OR 'zymfentra':ab,ti,kw | 164697 |
| #17 | 'janus kinase inhibitor'/exp | 37282 |
| #16 | 'interleukin 17'/exp | 74685 |
| #15 | 'tumor necrosis factor inhibitor'/exp | 133507 |
| #14 | 'filgotinib'/exp | 1470 |
| #13 | 'upadacitinib'/exp | 3357 |
| #12 | 'tofacitinib'/exp | 11664 |
| #11 | 'ixekizumab'/exp | 4211 |
| #10 | 'certolizumab pegol'/exp | 9941 |
| #9 | 'golimumab'/exp | 10946 |
| #8 | 'secukinumab'/exp | 8269 |
| #7 | 'bimekizumab'/exp | 690 |
| #6 | 'infliximab'/exp | 66519 |
| #5 | 'adalimumab'/exp | 49014 |
| #4 | 'etanercept'/exp | 39373 |
| #3 | #1 OR #2 | 41168 |
| #2 | 'ankyl* spond*litis':ab,ti,kw OR 'ankylo* spondyl*arthr*':ab,ti,kw OR 'ankylosing spine':ab,ti,kw OR 'be*hterew* disease':ab,ti,kw OR 'bekhterev disease':ab,ti,kw OR 'marie struempell disease':ab,ti,kw OR 'morbus bechterew':ab,ti,kw OR 'rheumatoid spondylitis':ab,ti,kw OR 'spin* ankylosis':ab,ti,kw OR 'spondyl* ankylopo*etica':ab,ti,kw OR 'spondylarthr*is ankylo*':ab,ti,kw OR 'vertebral ankylosis':ab,ti,kw | 30877 |
| #1 | 'ankylosing spondylitis'/exp | 35529 |

| Cochrane Library | | |
| --- | --- | --- |
| # |  |  |
| #1 | MeSH descriptor: [Spondylitis, Ankylosing] explode all trees | 929 |
| #2 | ('ankyl* spond*litis' OR 'ankylo* spondyl*arthr*' OR 'ankylosing spine' OR 'Be*hterew* disease' OR 'bekhterev disease' OR 'Marie Struempell Disease' OR 'morbus bechterew' OR 'Rheumatoid Spondylitis' OR 'spin* ankylosis' OR 'spondyl* ankylopo*etica' OR 'spondylarthr*is ankylo*' OR 'vertebral ankylosis'):ab,ti,kw | 2872 |
| #3 | #1 OR #2 | 2872 |
| #4 | MeSH descriptor: [Etanercept] explode all trees | 1018 |
| #5 | MeSH descriptor: [Adalimumab] explode all trees | 1164 |
| #6 | MeSH descriptor: [Infliximab] explode all trees | 1074 |
| #7 | MeSH descriptor: [Certolizumab Pegol] explode all trees | 220 |
| #8 | MeSH descriptor: [Tumor Necrosis Factor Inhibitors] explode all trees | 170 |
| #9 | MeSH descriptor: [Interleukin-17] explode all trees | 361 |
| #10 | MeSH descriptor: [Janus Kinase Inhibitors] explode all trees | 227 |
| #11 | ('abrilada' OR 'adalimumab' OR 'adaly' OR 'altebrel' OR 'amgevita' OR 'amjevita' OR 'amsparity' OR 'anti TNF' OR 'anti tumo*r necrosis factor agent' OR 'avakine' OR 'avent' OR 'avsola' OR 'benepali' OR 'bimekizumab' OR 'bimzelx' OR 'brenzys' OR 'certolizumab pegol' OR 'cimzia' OR 'cinnora' OR 'cosentyx' OR 'CTLA 8' OR 'CTLA8' OR 'cyltezo' OR 'Cytokine CX2' OR 'Cytokine ML 1' OR 'cytotoxic T lymphocyte antigen 8' OR 'cytotoxic T lymphocyte protein 8' OR 'D2E7 Antibody' OR 'davictrel' OR 'embrel' OR 'enbrel' OR 'enerceptan' OR 'erelzi' OR 'etacept' OR 'etanar' OR 'etanercept' OR 'eticovo' OR 'exemptia' OR 'filgotinib' OR 'flammegis' OR 'flixabi' OR 'fyzoclad' OR 'golimumab' OR 'hadlima' OR 'halimatoz' OR 'hefiya' OR 'hukyndra' OR 'hulio' OR 'humira' OR 'hyrimoz' OR 'idacio' OR 'IL 17*' OR 'imraldi' OR 'infimab' OR 'infinitam' OR 'inflectra' OR 'infliximab' OR 'interleukin 17*' OR 'Interleukin 25' OR 'ixekizumab' OR 'ixifi' OR 'JAK inhibitor*' OR 'Janus kinase inhibitor*' OR 'jaquinus' OR 'jyseleca' OR 'kromeya' OR 'libmyris' OR 'lifmior' OR 'MAb cA2' OR 'mabura' OR 'Monoclonal Antibody cA2' OR 'monoclonal antibody D2E7' OR 'nepexto' OR 'opinercept' OR 'pegylated tumo*r necrosis factor alpha antibody Fab fragment' OR 'qletli' OR 'raheara' OR 'remicade' OR 'remsima' OR 'renflexis' OR 'reumatocept' OR 'revellex' OR 'rinvoq' OR 'scapho' OR 'secukinumab' OR 'shinponi' OR 'simponi' OR 'simziya' OR 'solymbic' OR 'sulinno' OR 'taltz' OR 'tasocitinib' OR 'TNF a' OR 'TNF Antagonist*' OR 'TNF Blocker*' OR 'TNF inhibitor*' OR 'TNF Receptor Type II IgG Fusion Protein' OR 'TNFR Fc Fusion Protein' OR 'TNT Receptor Fusion Protein' OR 'TNTR Fc' OR 'tofacitinib' OR 'trudexa' OR 'tumo*r necrosis factor inhibitor*' OR 'tumo*r necrosis factor receptor Fc fusion protein' OR 'tumor necrosis factor alpha inhibitor' OR 'Tumor Necrosis Factor Antagonist' OR 'Tumor Necrosis Factor Blocker*' OR 'tunex' OR 'upadacitinib' OR 'xcimzane' OR 'xeljanz' OR 'yisaipu' OR 'yuflyma' OR 'yusimry' OR 'zessly' OR 'zymfentra'):ab,ti,kw | 36297 |
| #12 | #4 OR #5 OR #6 OR #7 OR #8 OR #9 OR #10 OR #11 | 36297 |
| #13 | #3 AND #12 | 1623 |

| Web of Science | | |
| --- | --- | --- |
| # | Query | Results |
| #1 | TS=((ankyl* spond*litis) OR (ankylo* spondyl*arthr*) OR (ankylosing spine) OR (Be*hterew* disease) OR (bekhterev disease) OR (Marie Struempell Disease) OR (morbus bechterew) OR (Rheumatoid Spondylitis) OR (spin* ankylosis) OR (spondyl* ankylopo*etica) OR (spondylarthr*is ankylo*) OR (vertebral ankylosis)) | 27684 |
| #2 | TS=((abrilada) OR (adalimumab) OR (adaly) OR (altebrel) OR (amgevita) OR (amjevita) OR (amsparity) OR (anti TNF) OR (anti tumo*r necrosis factor agent) OR (avakine) OR (avent) OR (avsola) OR (benepali) OR (bimekizumab) OR (bimzelx) OR (brenzys) OR (certolizumab pegol) OR (cimzia) OR (cinnora) OR (cosentyx) OR (CTLA 8) OR (CTLA8) OR (cyltezo) OR (Cytokine CX2) OR (Cytokine ML 1) OR (cytotoxic T lymphocyte antigen 8) OR (cytotoxic T lymphocyte protein 8) OR (D2E7 Antibody) OR (davictrel) OR (embrel) OR (enbrel) OR (enerceptan) OR (erelzi) OR (etacept) OR (etanar) OR (etanercept) OR (eticovo) OR (exemptia) OR (filgotinib) OR (flammegis) OR (flixabi) OR (fyzoclad) OR (golimumab) OR (hadlima) OR (halimatoz) OR (hefiya) OR (hukyndra) OR (hulio) OR (humira) OR (hyrimoz) OR (idacio) OR (IL-17*) OR (imraldi) OR (infimab) OR (infinitam) OR (inflectra) OR (infliximab) OR (interleukin-17*) OR (Interleukin 25) OR (ixekizumab) OR (ixifi) OR (JAK inhibitor*) OR (Janus kinase inhibitor*) OR (jaquinus) OR (jyseleca) OR (kromeya) OR (libmyris) OR (lifmior) OR (MAb cA2) OR (mabura) OR (Monoclonal Antibody cA2) OR (monoclonal antibody D2E7) OR (nepexto) OR (opinercept) OR (pegylated tumo*r necrosis factor alpha antibody Fab fragment) OR (qletli) OR (raheara) OR (remicade) OR (remsima) OR (renflexis) OR (reumatocept) OR (revellex) OR (rinvoq) OR (scapho) OR (secukinumab) OR (shinponi) OR (simponi) OR (simziya) OR (solymbic) OR (sulinno) OR (taltz) OR (tasocitinib) OR (TNF a) OR (TNF Antagonist*) OR (TNF Blocker*) OR (TNF inhibitor*) OR (TNF Receptor Type II IgG Fusion Protein) OR (TNFR Fc Fusion Protein) OR (TNT Receptor Fusion Protein) OR (TNTR Fc) OR (tofacitinib) OR (trudexa) OR (tumo*r necrosis factor inhibitor*) OR (tumo*r necrosis factor receptor Fc fusion protein) OR (tumor necrosis factor alpha inhibitor) OR (Tumor Necrosis Factor Antagonist) OR (Tumor Necrosis Factor Blocker*) OR (tunex) OR (upadacitinib) OR (xcimzane) OR (xeljanz) OR (yisaipu) OR (yuflyma) OR (yusimry) OR (zessly) OR (zymfentra)) | 400747 |
| #3 | #2 AND #1 | 7145 |
