# Supplementary material for: Risk of new-onset and recurrent uveitis with different biologics for ankylosing spondylitis: a network meta-analysis
Source: Front Immunol. 2025 Jun 20;16:1556313. doi: 10.3389/fimmu.2025.1556313 (PMC12226306; doi:10.3389/fimmu.2025.1556313)
Supplement: Supplementary file 3 [file Table3.docx]

**Details of heterogeneity test**


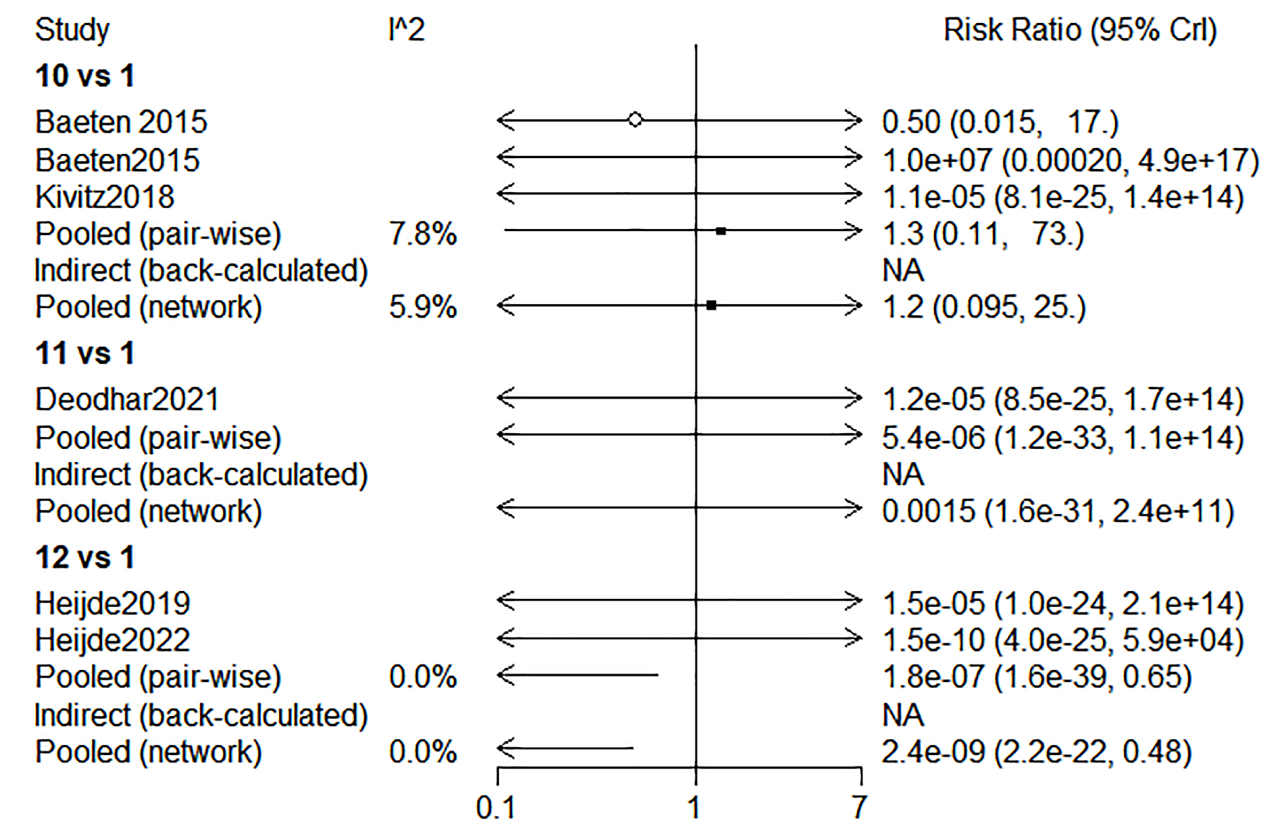
Figure S1 Results of heterogeneity testing for new-onset uveitis


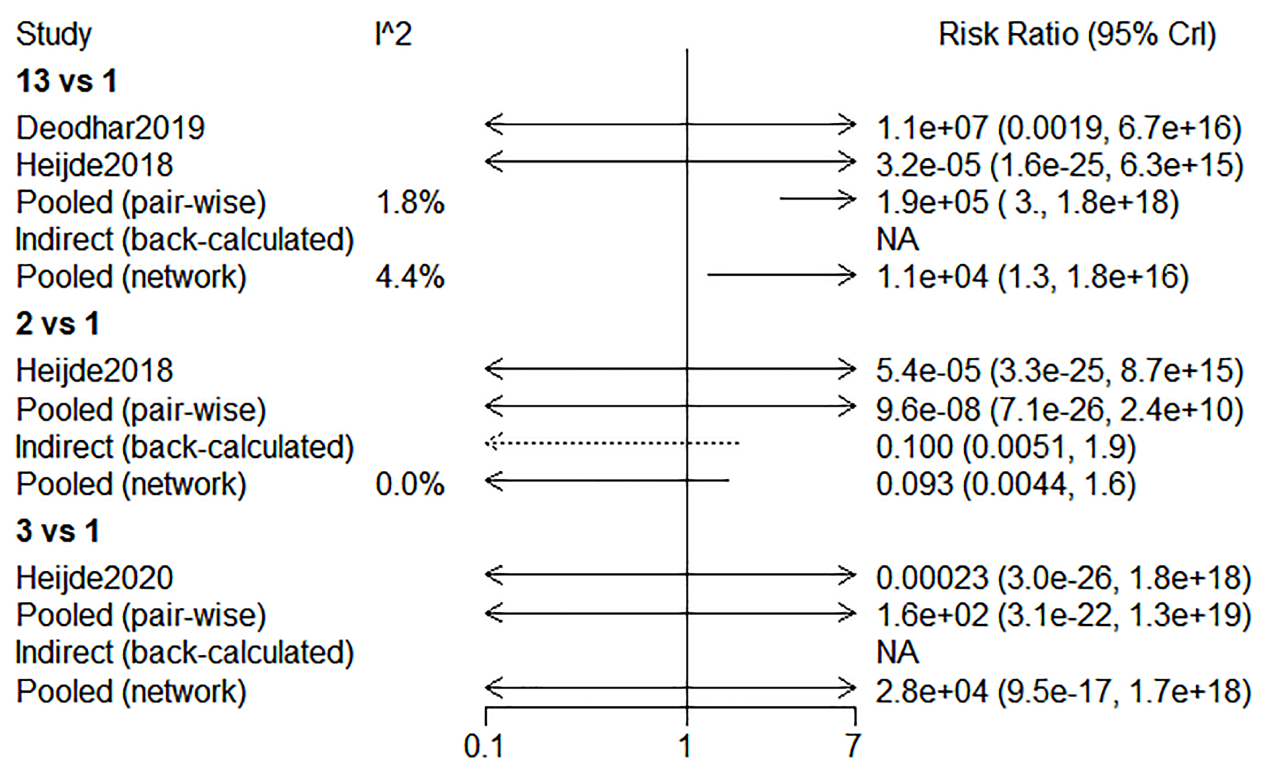
Figure S2 Results of heterogeneity testing for new-onset uveitis


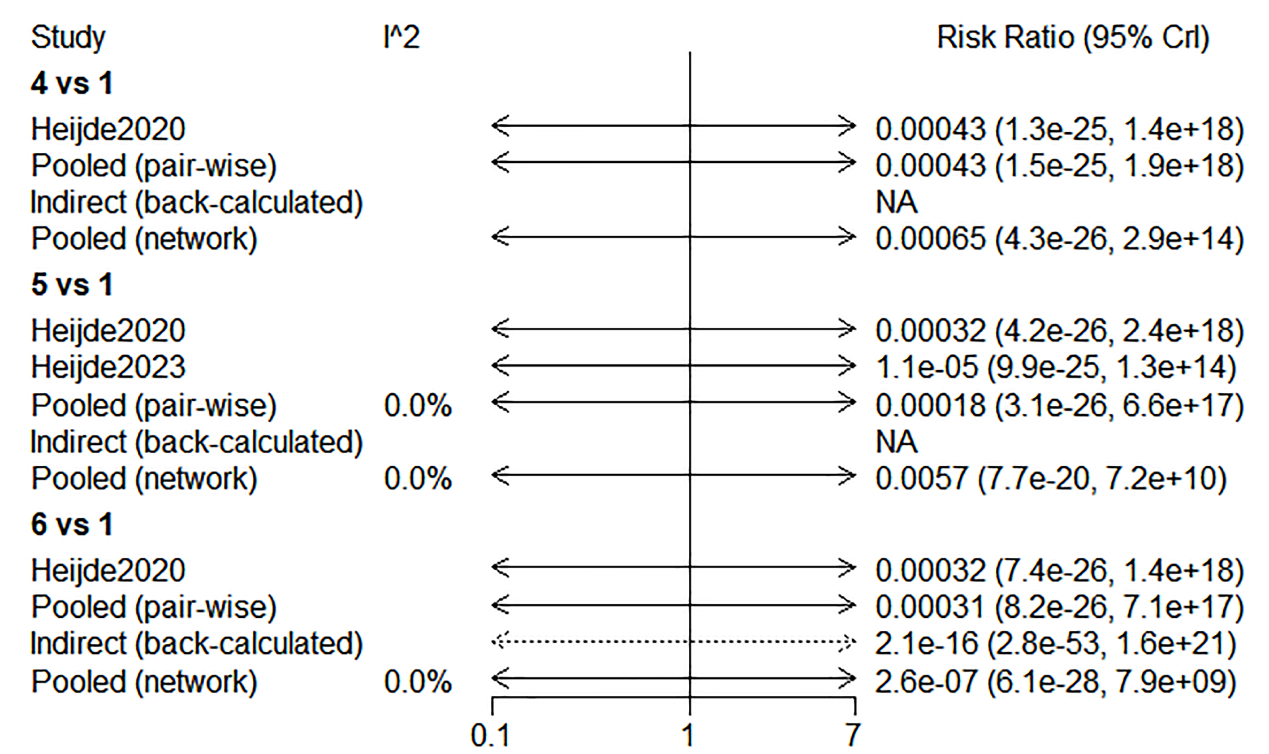
Figure S3 Results of heterogeneity testing for new-onset uveitis


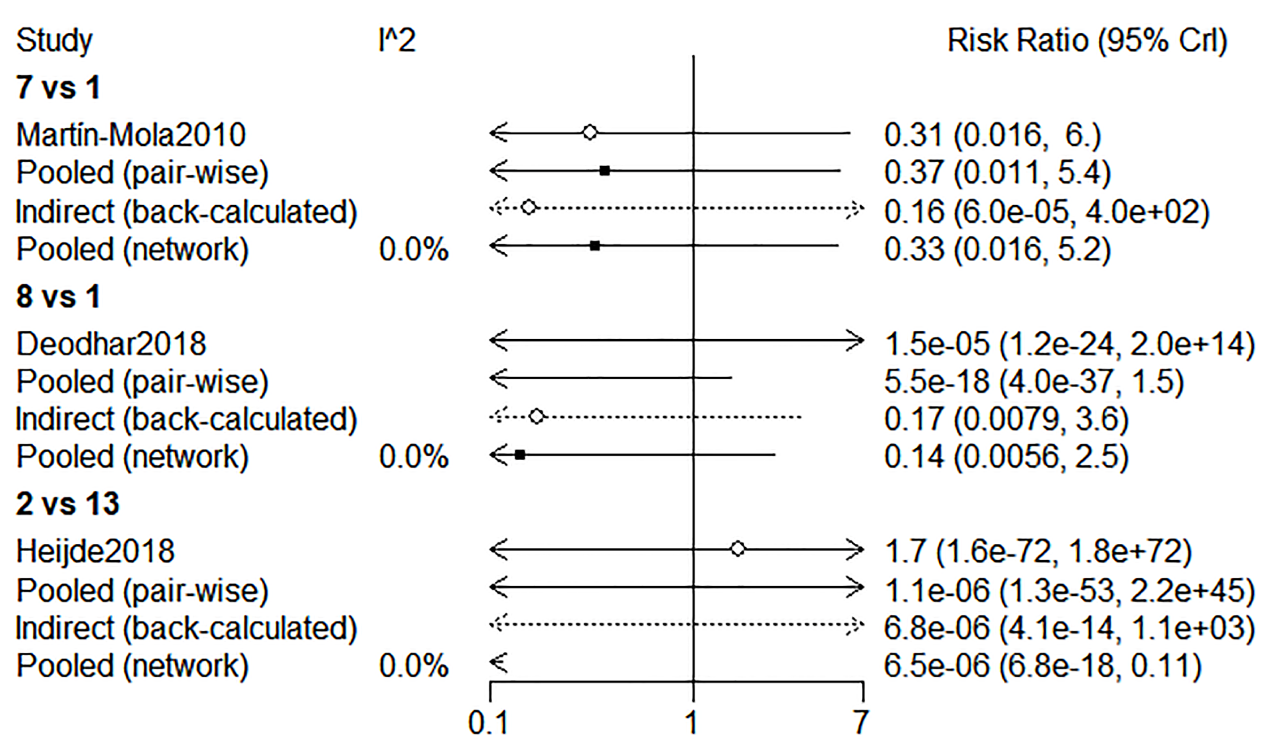
Figure S4 Results of heterogeneity testing for new-onset uveitis


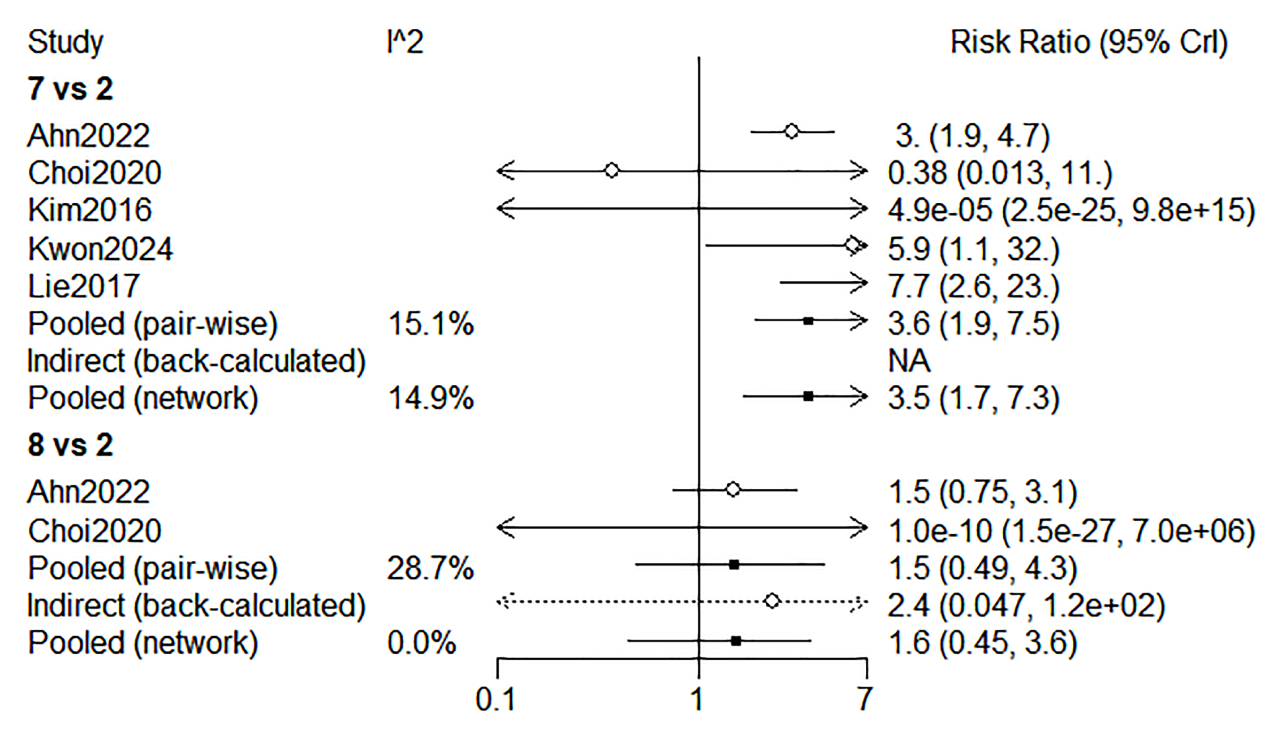
Figure S5 Results of heterogeneity testing for new-onset uveitis


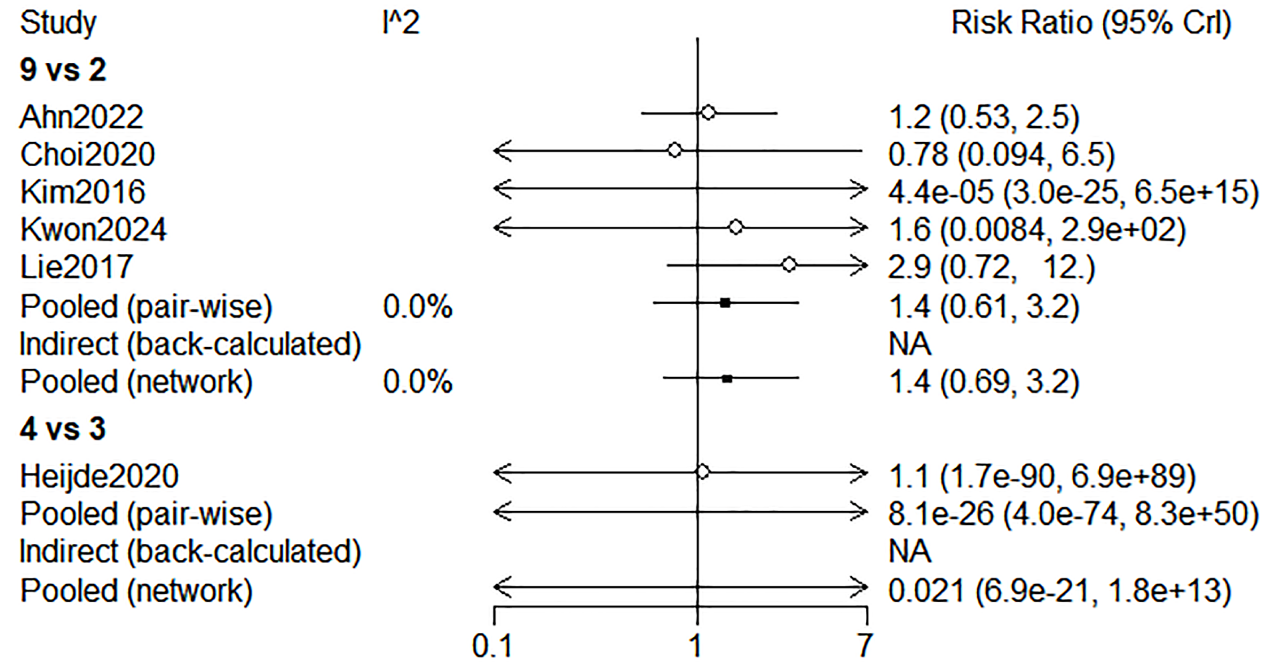


Figure S6 Results of heterogeneity testing for new-onset uveitis


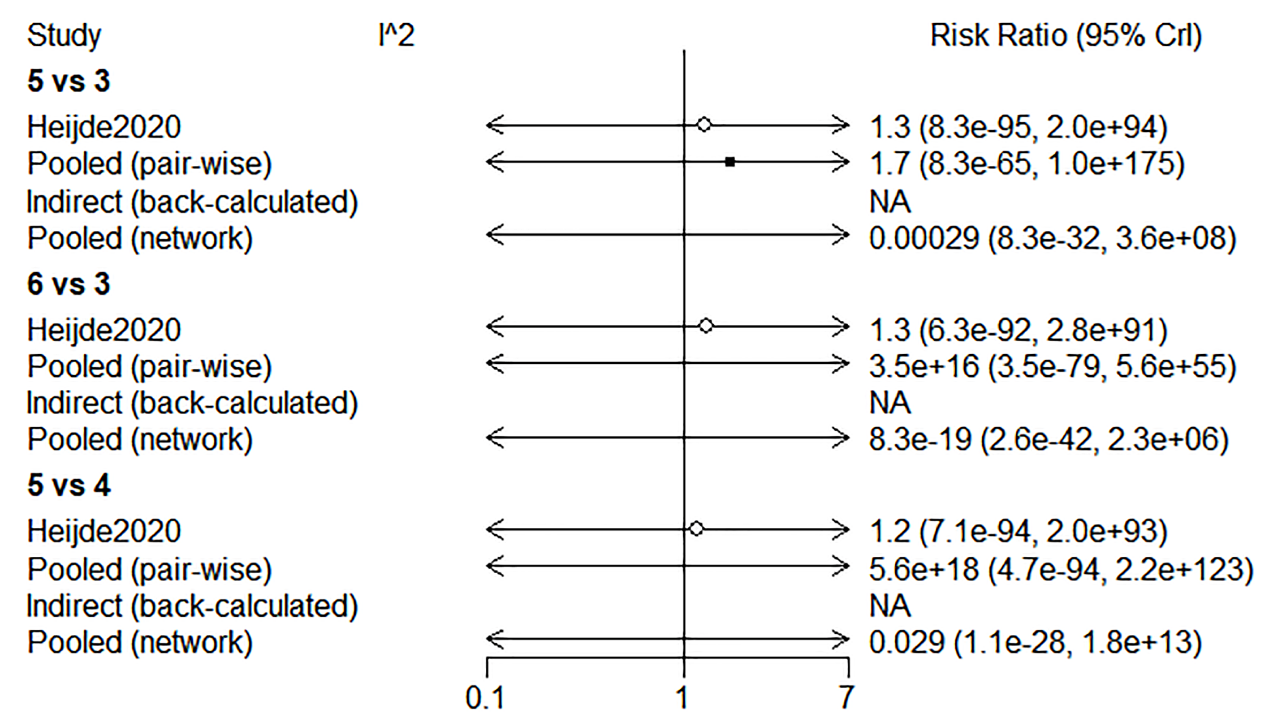
Figure S7 Results of heterogeneity testing for new-onset uveitis


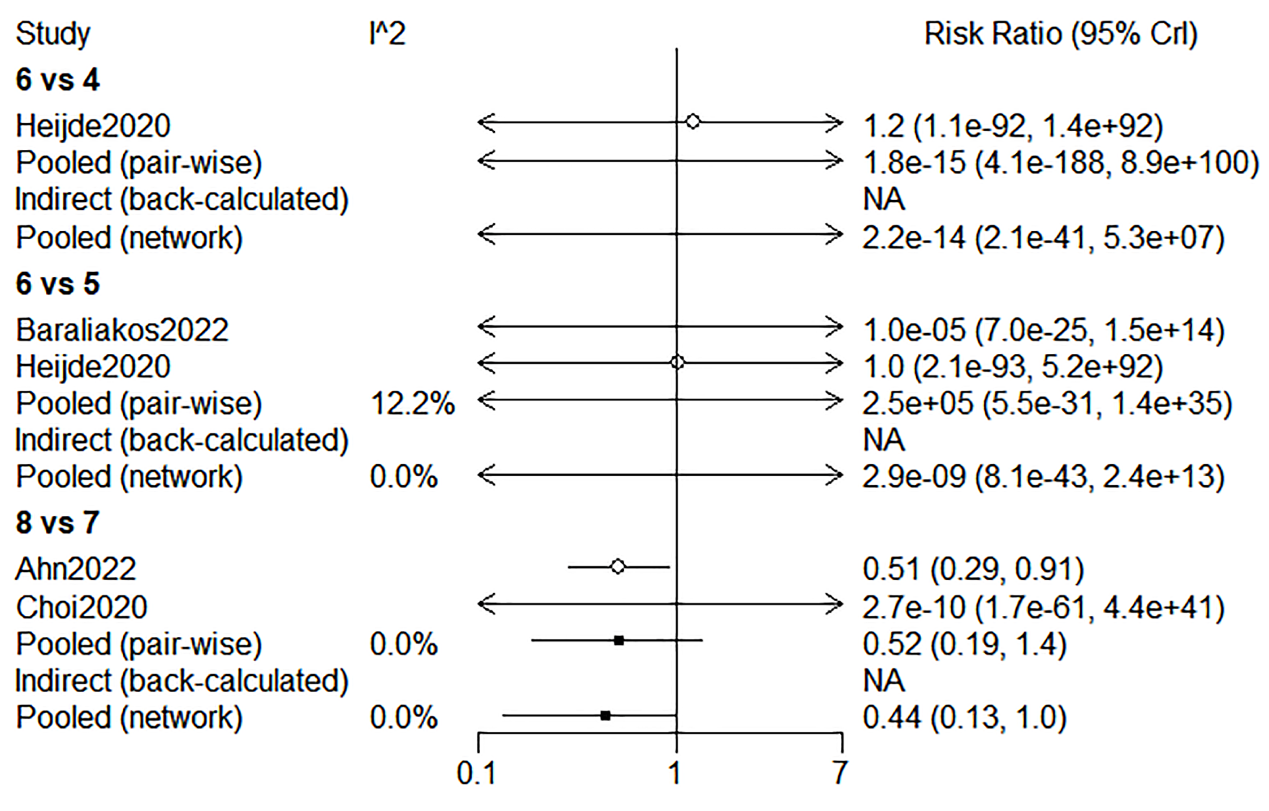
Figure S8 Results of heterogeneity testing for new-onset uveitis


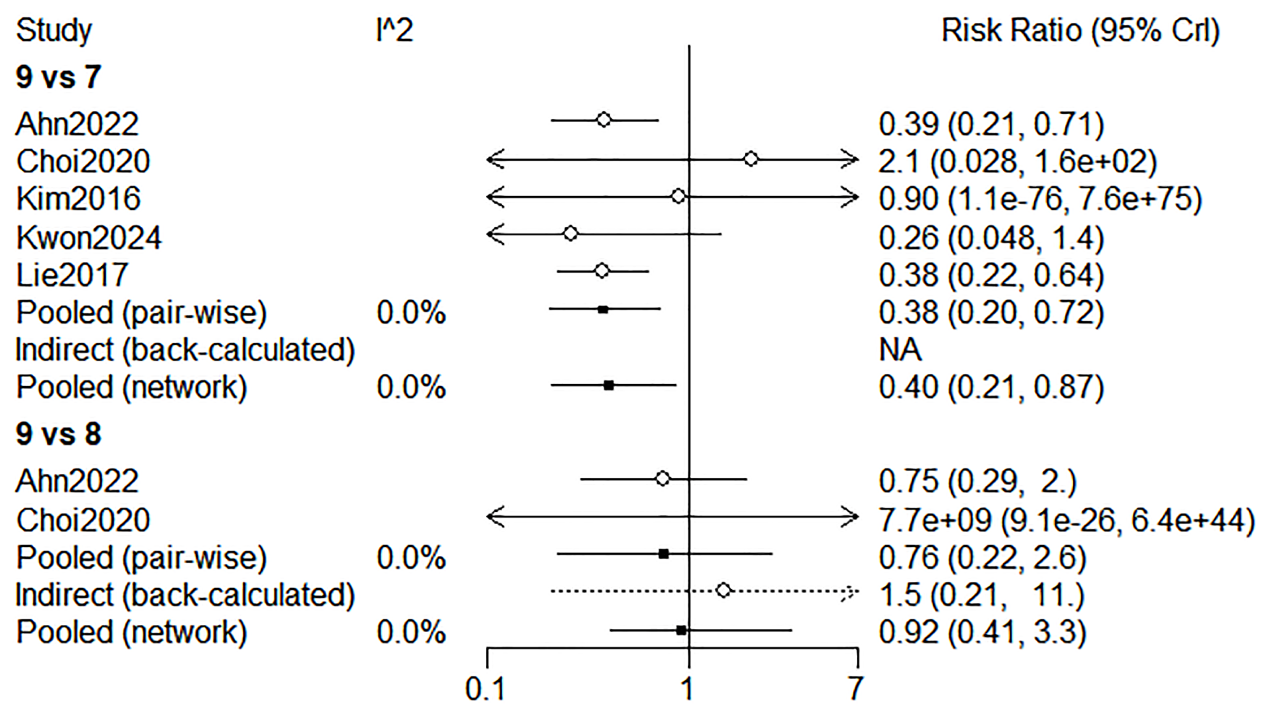
Figure S9 Results of heterogeneity testing for new-onset uveitis


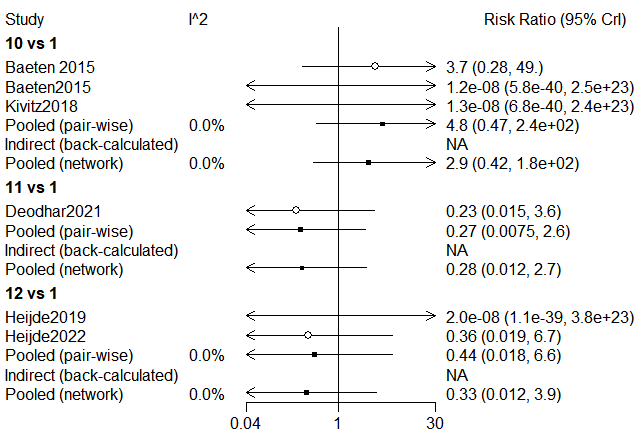
Figure S10 Results of heterogeneity testing for recurrent uveitis


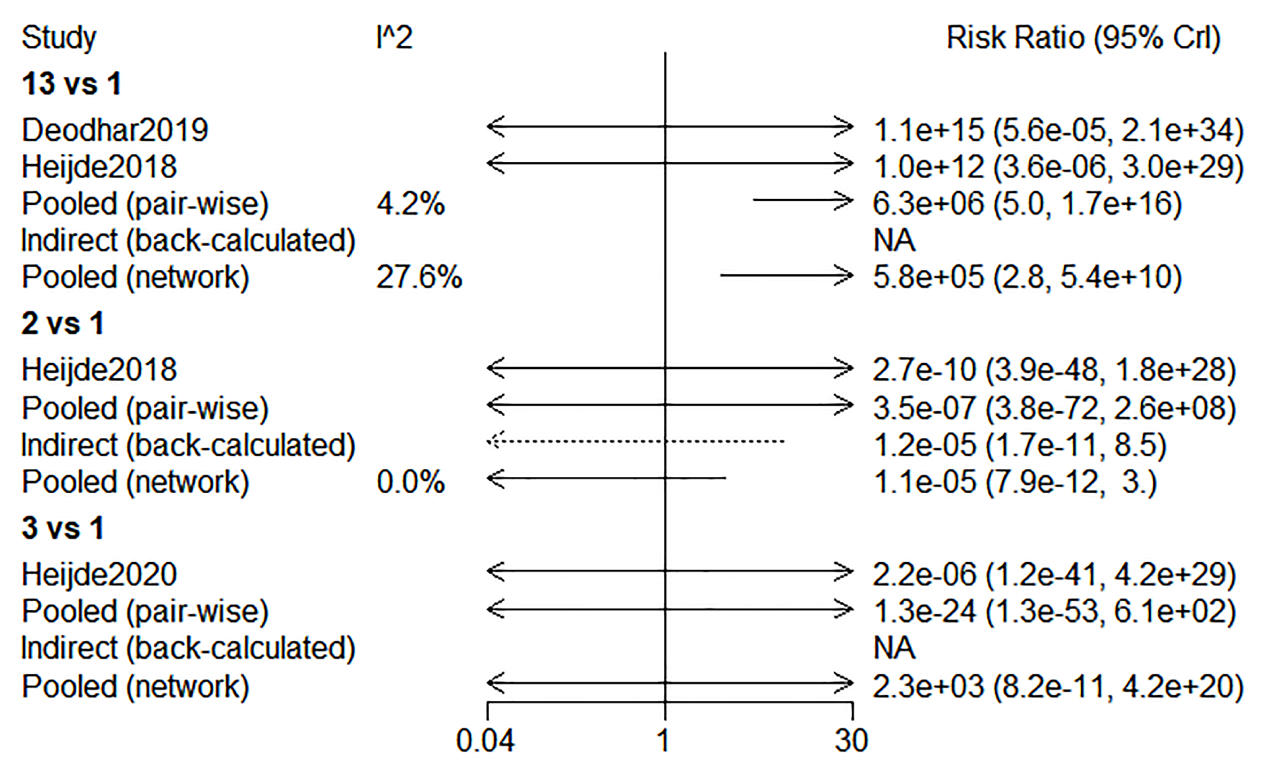
Figure S11 Results of heterogeneity testing for recurrent uveitis


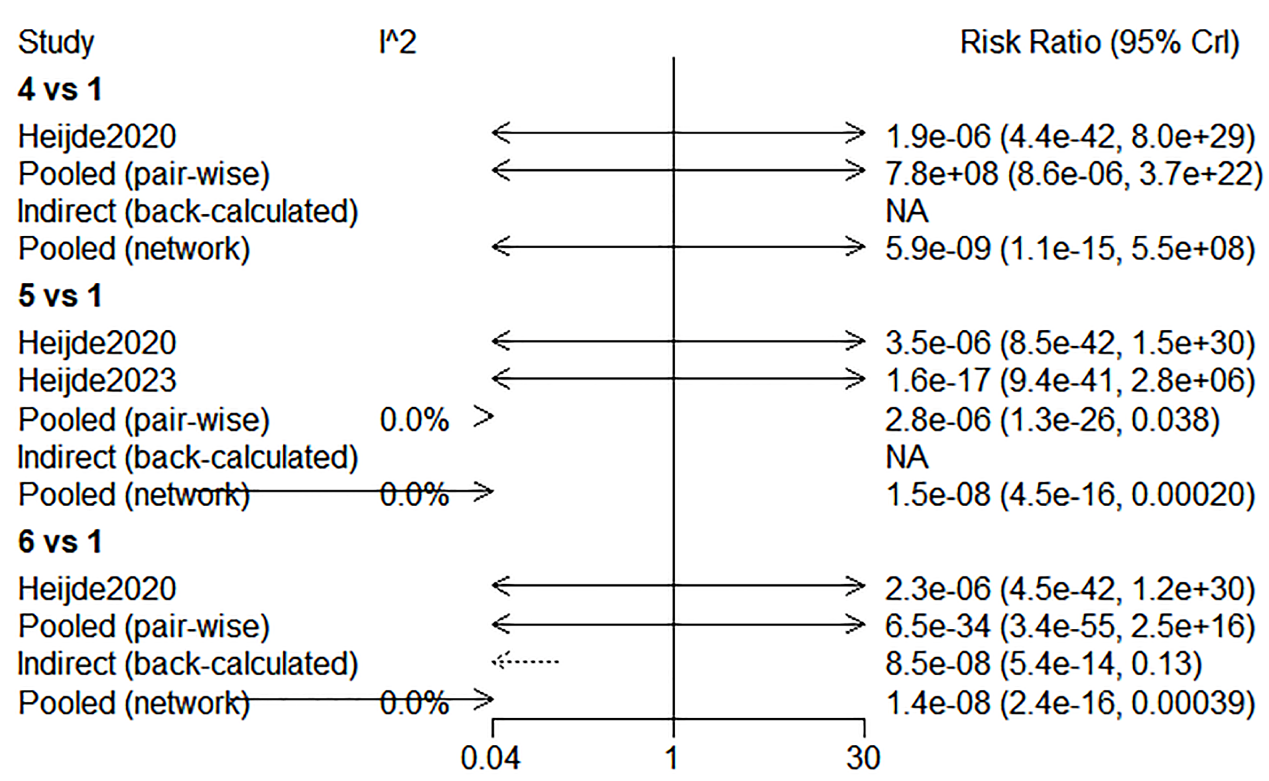
Figure S12 Results of heterogeneity testing for recurrent uveitis


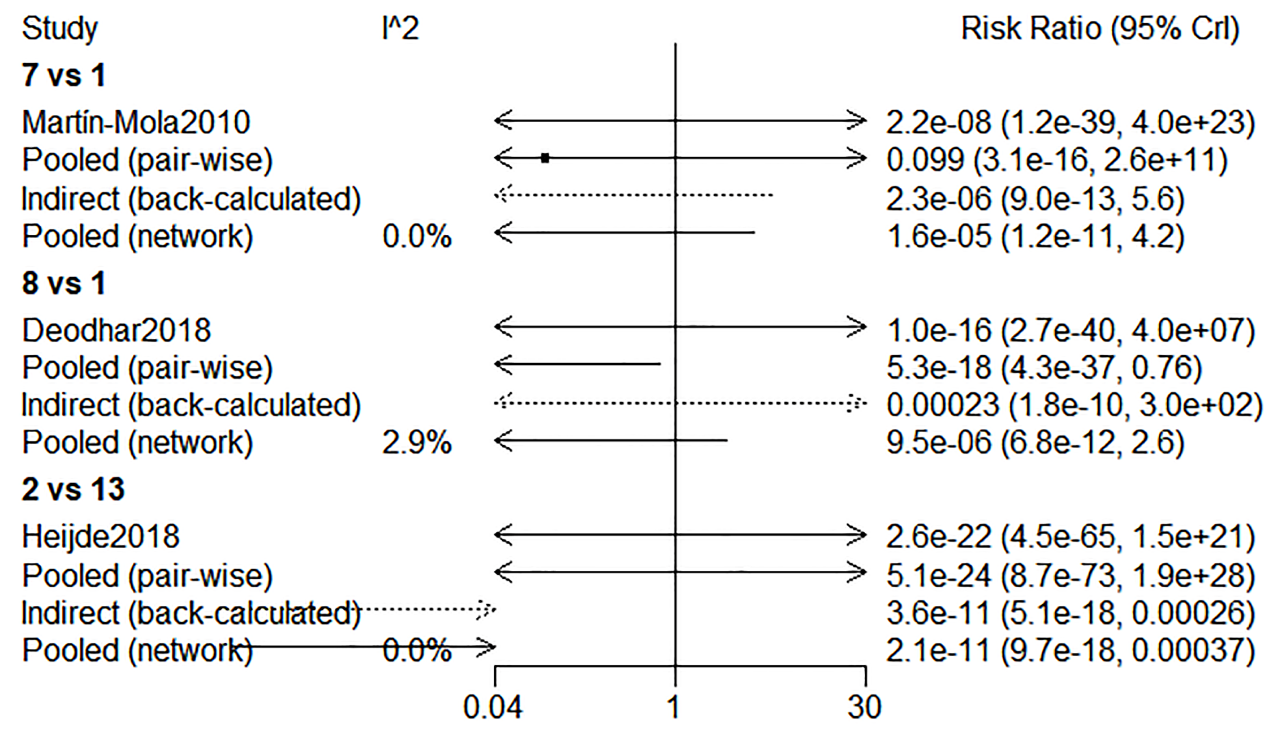
Figure S13 Results of heterogeneity testing for recurrent uveitis


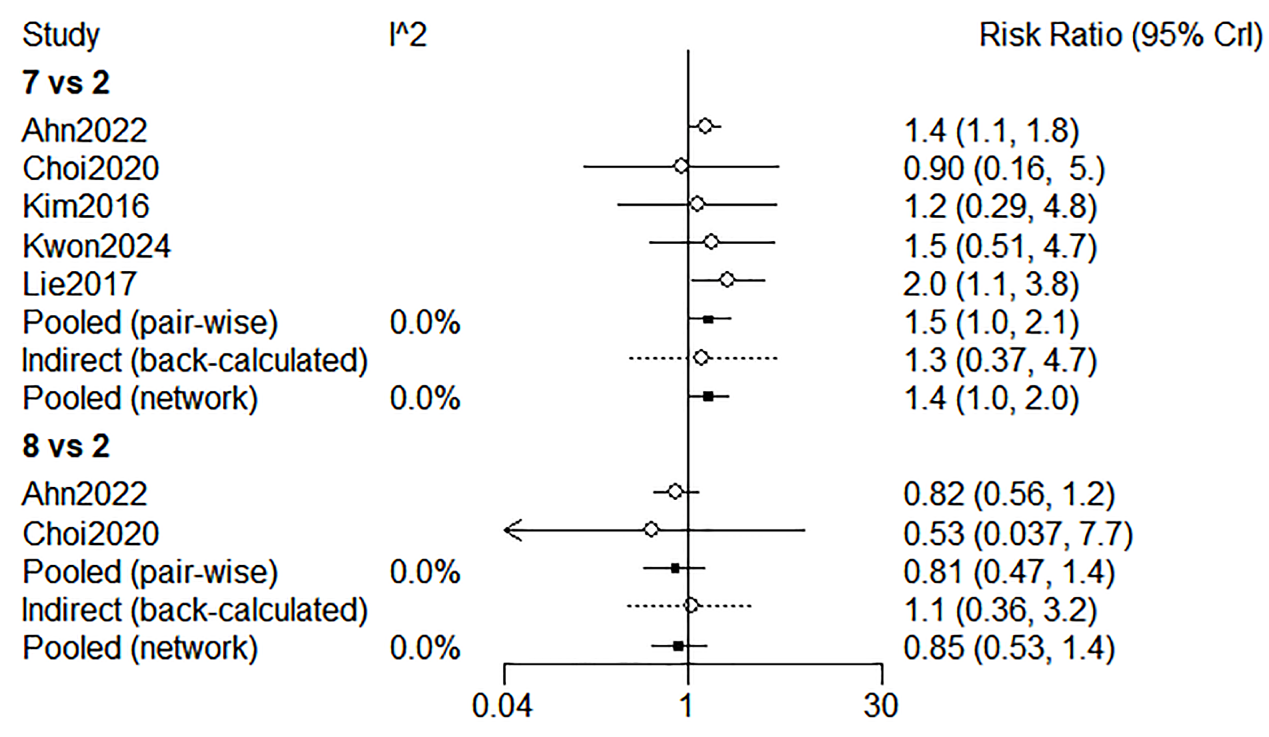
Figure S14 Results of heterogeneity testing for recurrent uveitis


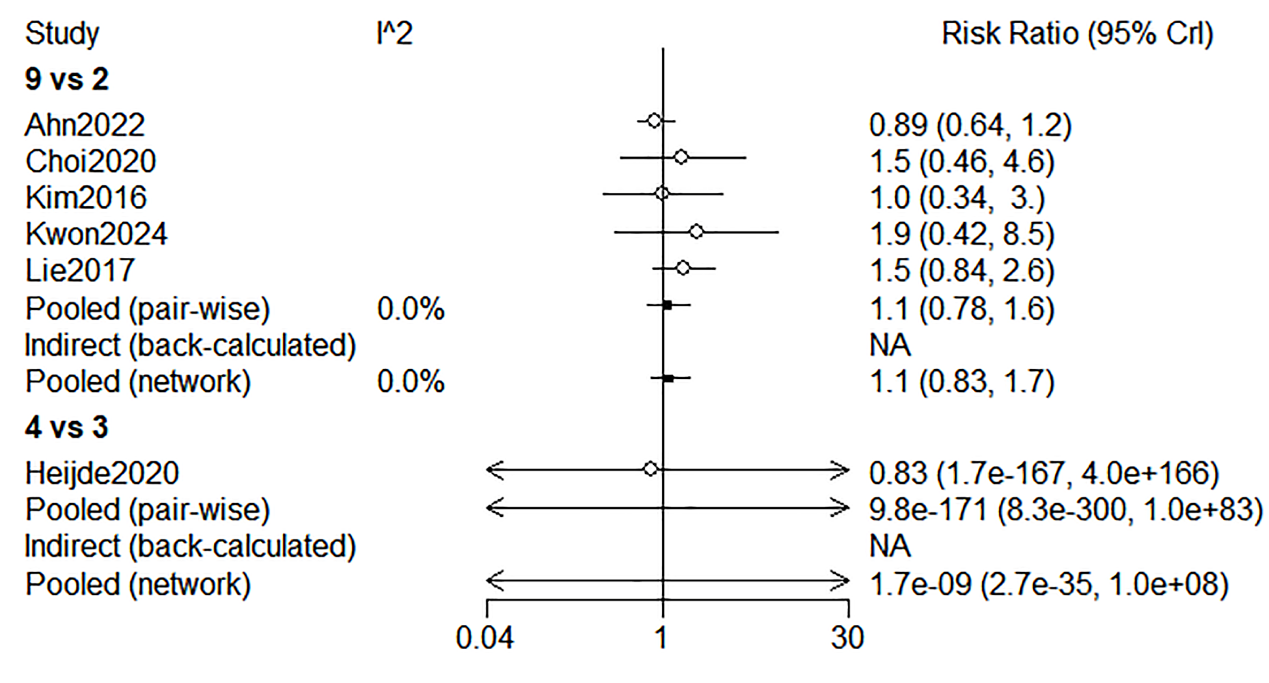
Figure S15 Results of heterogeneity testing for recurrent uveitis


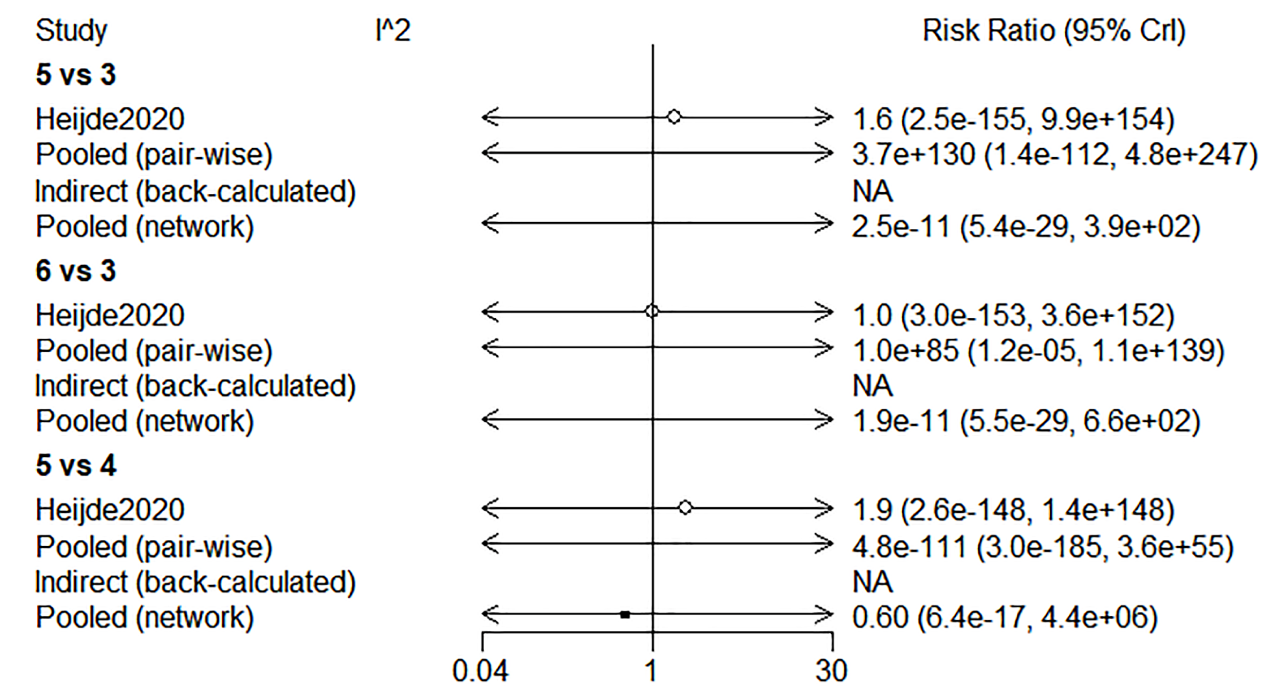
Figure S16 Results of heterogeneity testing for recurrent uveitis


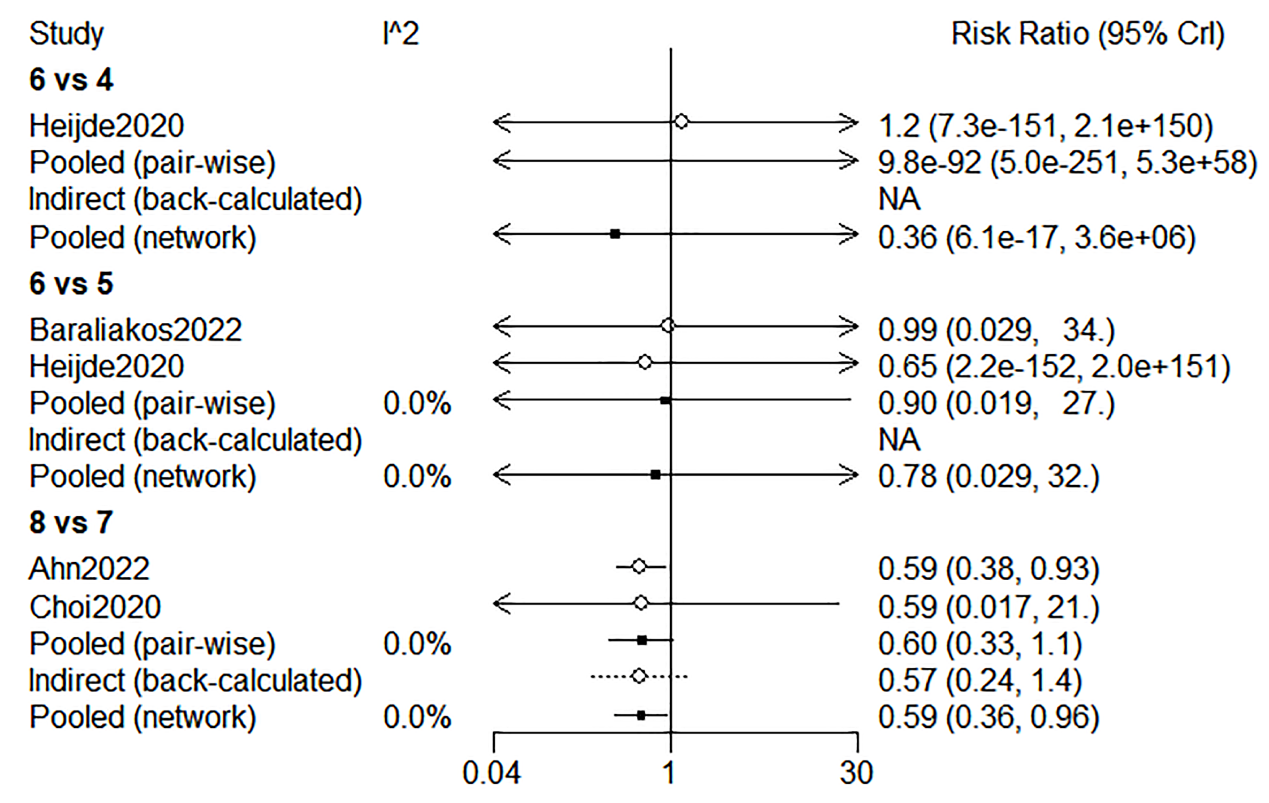
Figure S17 Results of heterogeneity testing for recurrent uveitis


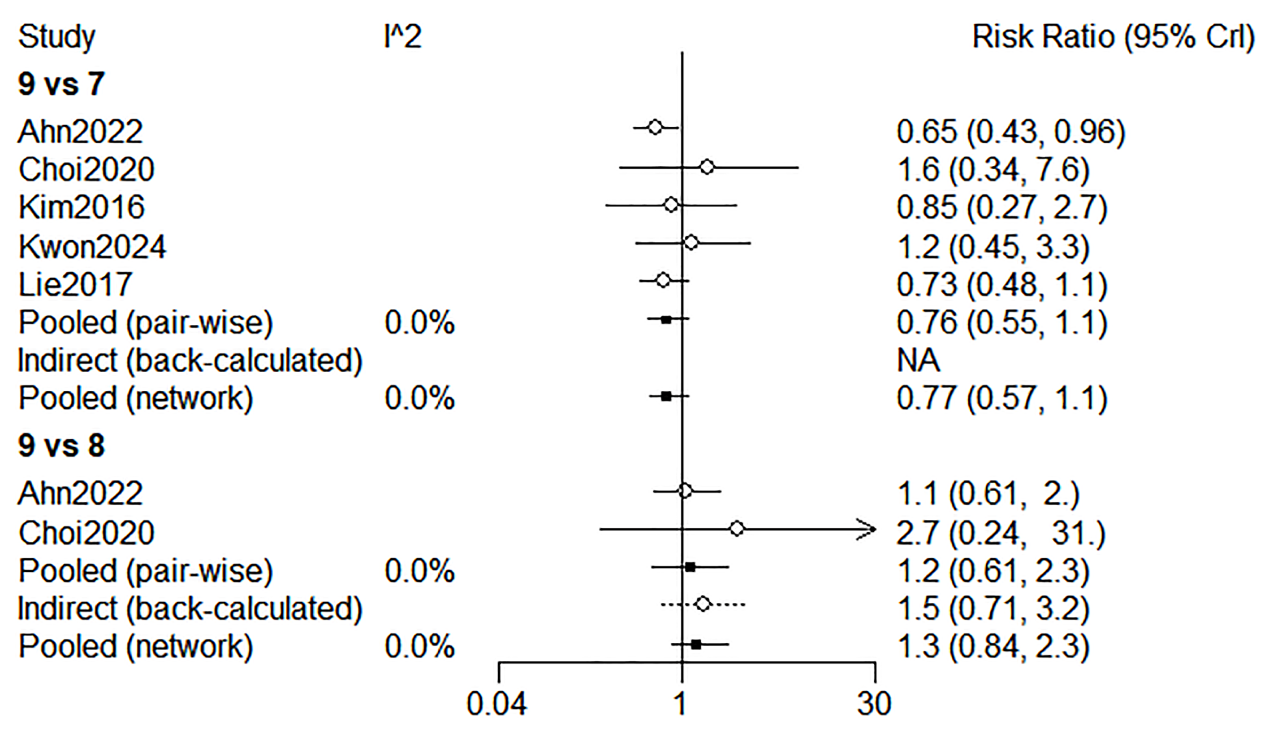
Figure S18 Results of heterogeneity testing for recurrent uveitis
