# Supplementary material for: Risk of new-onset and recurrent uveitis with different biologics for ankylosing spondylitis: a network meta-analysis
Source: Front Immunol. 2025 Jun 20;16:1556313. doi: 10.3389/fimmu.2025.1556313 (PMC12226306; doi:10.3389/fimmu.2025.1556313)

1. Forest Plot of Consistency Test for Recurrent Uveitis


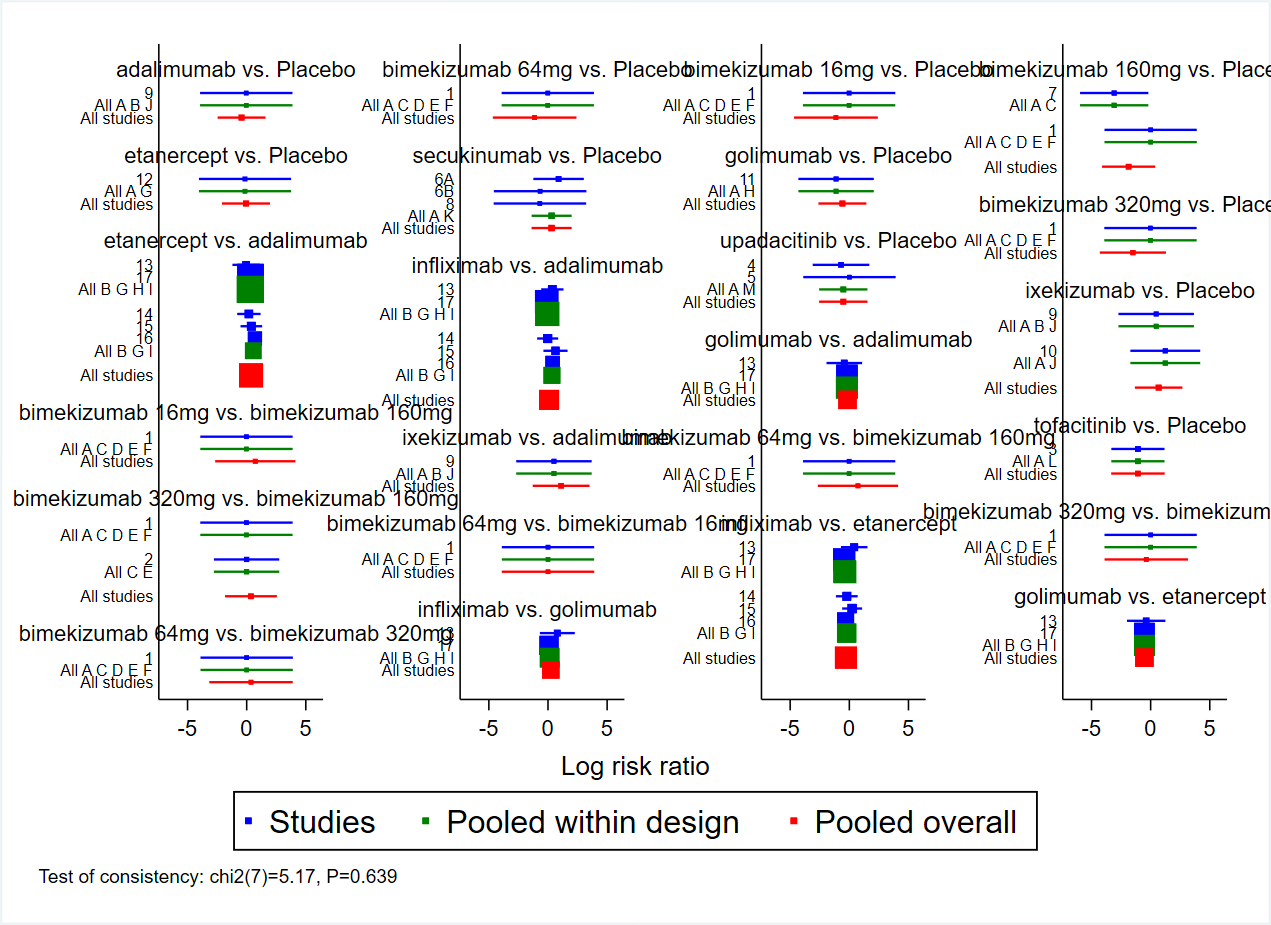


1. Forest Plot of Consistency Test for New-onset Uveitis


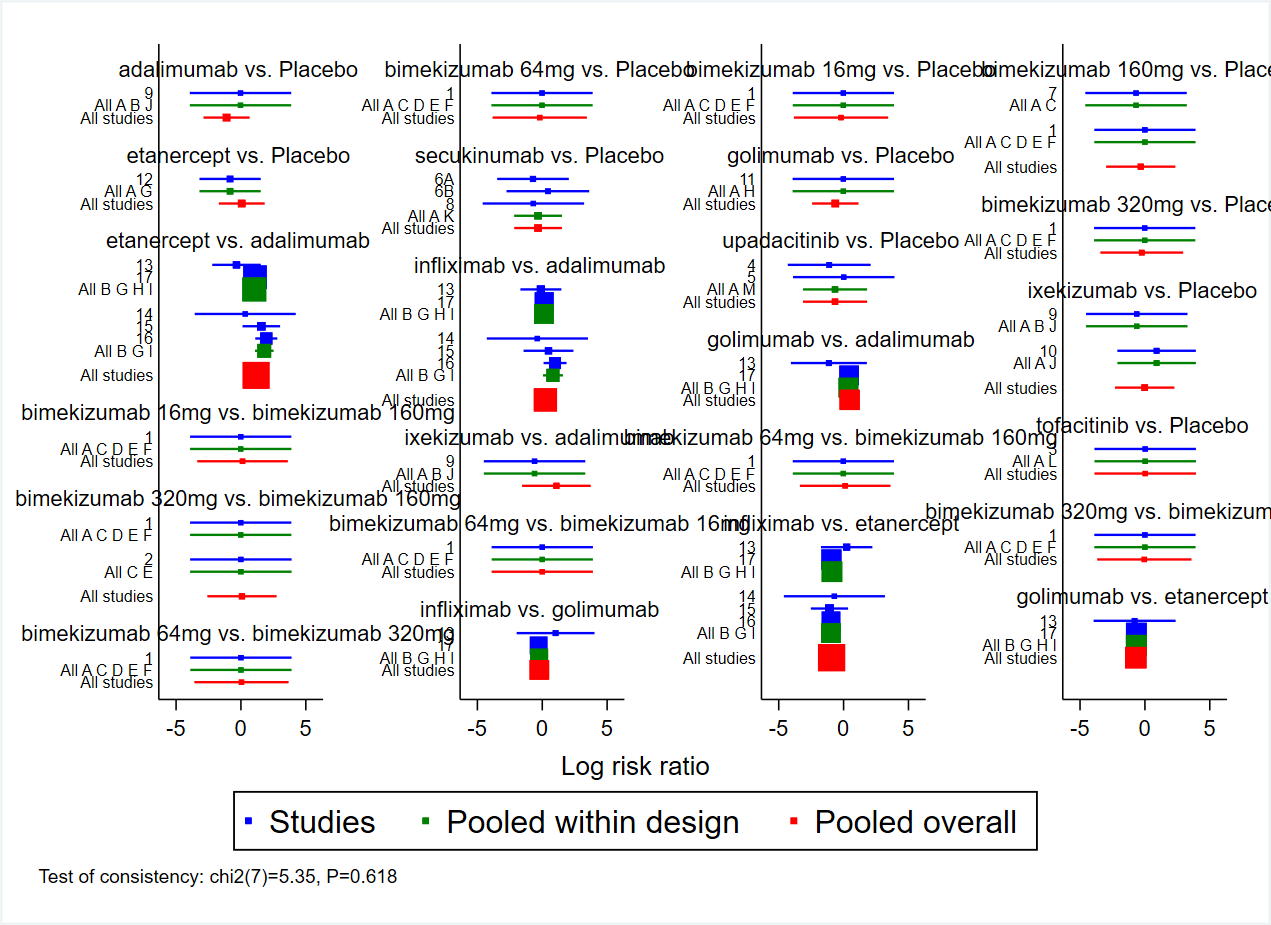

Supplement: Supplementary file 4 [file Table4.doc]
